# Structure of 1,5-benzodiazepinones in the solid state and in solution: Effect of the fluorination in the six-membered ring

**DOI:** 10.3762/bjoc.9.253

**Published:** 2013-10-21

**Authors:** Marta Pérez-Torralba, Rosa M Claramunt, M Ángeles García, Concepción López, M Carmen Torralba, M Rosario Torres, Ibon Alkorta, José Elguero

**Affiliations:** 1 Departamento de Química Orgánica y Bio-Orgánica, Facultad de Ciencias, UNED, Paseo Senda del Rey 9, 28040-Madrid, Spain; 2 Departamento de Química Inorgánica I and CAI de Difracción de Rayos-X, Facultad de Ciencias Químicas, UCM, 28040-Madrid, Spain; 3 Instituto de Química Médica, Centro de Química Orgánica Manuel Lora-Tamayo, IQM-CSIC, Juan de la Cierva 3, 28006-Madrid, Spain

**Keywords:** benzodiazepinones, DFT, GIAO calculations, inversion barriers, multinuclear NMR, tautomerism, X-ray structures

## Abstract

Two novel tetrafluorinated 1,5-benzodiazepinones were synthesized and their X-ray structures determined. 6,7,8,9-Tetrafluoro-4-methyl-1,3-dihydro-2*H*-1,5-benzodiazepin-2-one crystallizes in the monoclinic *P*2_1_/*c* space group and 6,7,8,9-tetrafluoro-1,4-dimethyl-1,3-dihydro-2*H*-1,5-benzodiazepin-2-one in the triclinic *P*−1 space group. Density functional theory studies at the B3LYP/6-311++G(d,p) level were carried out on these compounds and on four non-fluorinated derivatives, allowing to calculate geometries, tautomeric energies and ring-inversion barriers, that were compared with the experimental results obtained by static and dynamic NMR in solution and in solid state.

## Introduction

**Figure 1:**
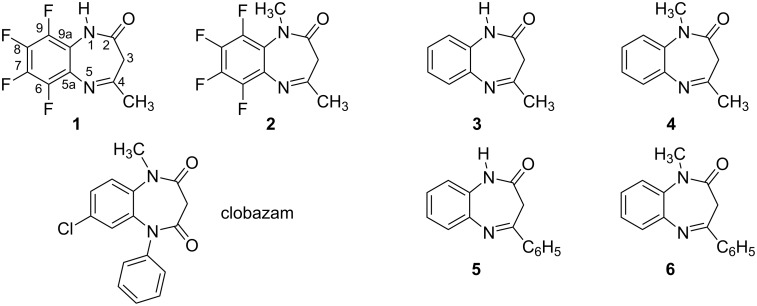
The six 1,5-benzodiazepinones discussed in this paper together with clobazam.

In our previous paper [1] we already reported the relevance of 1,5-benzodiazepine derivatives in central nervous system pathologies as well as for other applications in medicinal chemistry [2–6], the most important is clobazam (7-chloro-1-methyl-5-phenyl-1*H*-1,5-benzodiazepine-2,4(3*H*,5*H*)-dione, Figure 1). As a continuation of our research program on the synthesis, spectroscopic and biological properties of 1,5-benzodiazepine derivatives as well as their calculated parameters, we report in the present publication the experimental and theoretical studies of 1,5-benzodiazepinones **1**–**6**; note that only compounds **1** and **2** are new; for compounds **3**–**6** we used literature data together with new computational results.

## Results and Discussion

### Synthesis

**Scheme 1:**
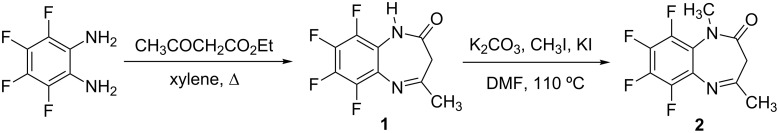
Synthesis of compounds **1** and **2**.

6,7,8,9-Tetrafluoro-4-methyl-1,3-dihydro-2*H*-1,5-benzodiazepin-2-one (**1**) was prepared in 63% yield by the reaction of 1,2-diamino-3,4,5,6-tetrafluorobenzene with ethyl acetylacetate (ethyl 3-oxobutanoate) following the literature procedure to prepare **3** [7] (Scheme 1). Further treatment with iodomethane under basic conditions afforded 6,7,8,9-tetrafluoro-1,4-dimethyl-1,3-dihydro-2*H*-1,5-benzodiazepin-2-one (**2**) in 80% yield.

### Geometries

**Figure 2:**
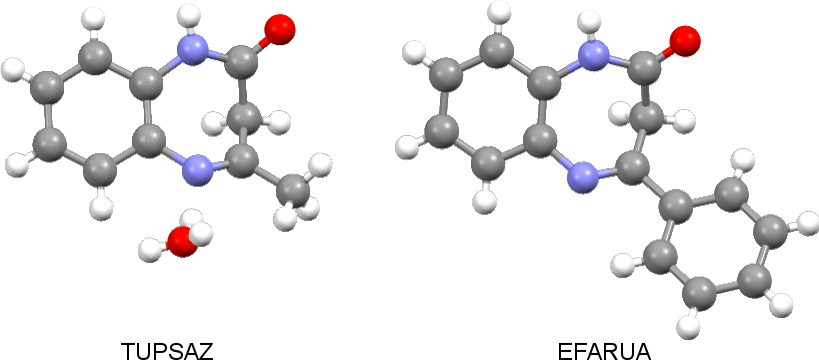
The X-ray structures of **3a** (TUPSAZ), **5a** (EFARUA). In TUPSAZ there is a disordered water molecule.

The geometries of two related structures together with their codes as reported in the Cambridge Structural Database [8–9] are shown in Figure 2.

**Figure 3:**
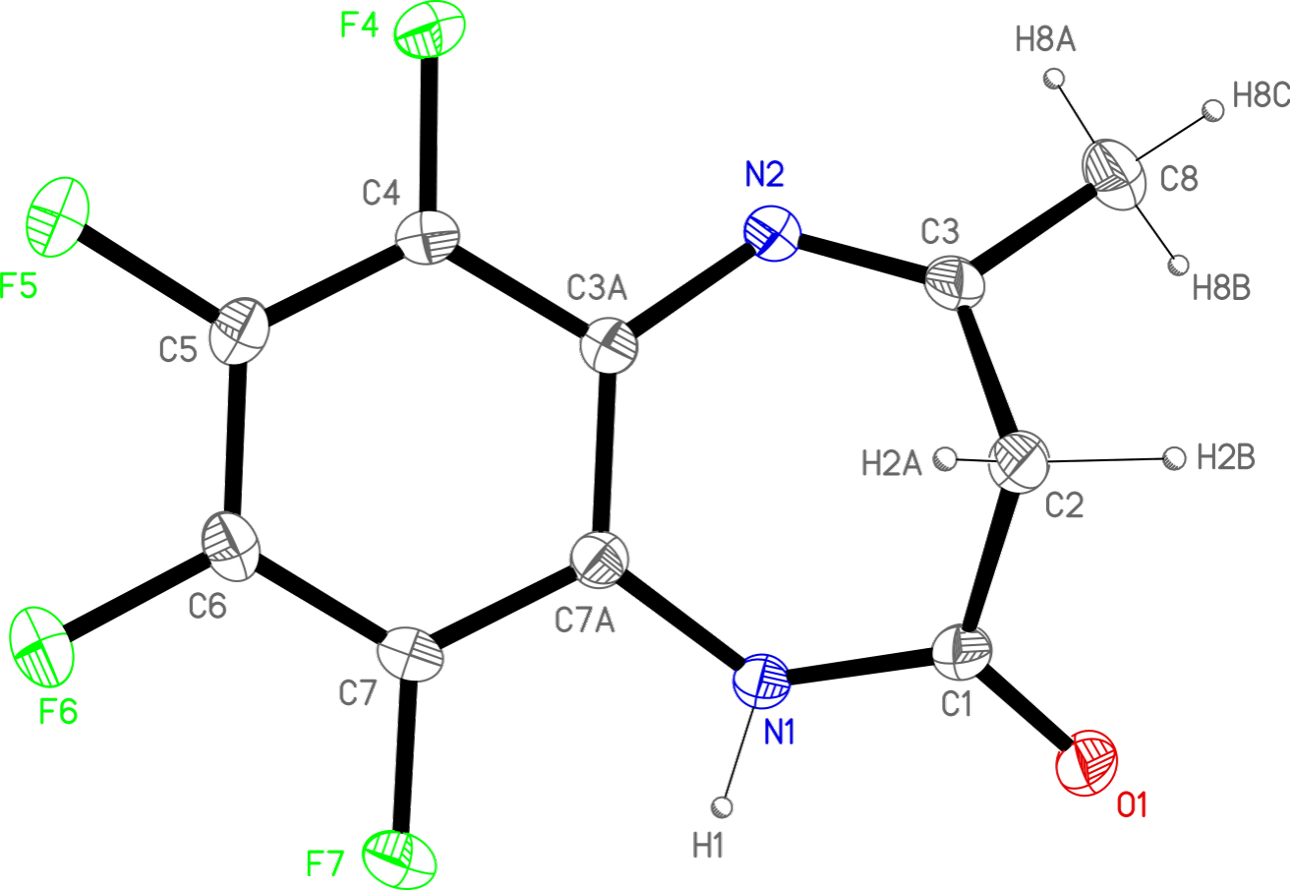
ORTEP plot (30% probability) of **1**, showing the X-ray labeling of the asymmetric unit.

Compound **1** crystallizes in the monoclinic *P*2_1_/*c* space group containing one molecule per asymmetric unit (Figure 3; the numbering used in the crystallographic part is different from that of Figure 1). The bonding distances and angles agree with the electronic distribution according to one amide group on the C1 atom and one double bond, C3–N2, (tautomer **a** in Figure 7). The molecule is not planar due to the folding of the seven-membered ring with C1, C2 and C3 out of the plane defined by the aromatic ring and the nitrogen atoms. The dihedral angles between this plane and those formed by the C1N1O1 and N2C3C8 atoms are 35.4(2)º and 41.7(2)º, respectively.

**Figure 4:**
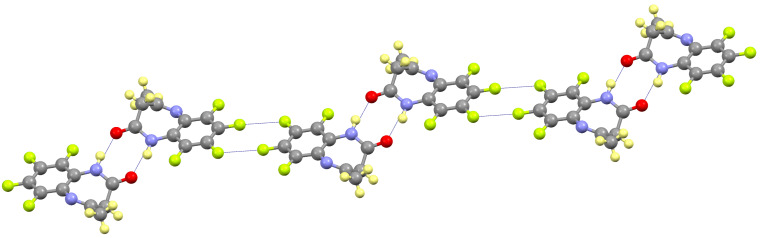
View of the *zigzag* chain formed in **1**, showing the H-bond and F–F interactions.

These molecules are linked forming dimers by symmetric hydrogen bonds between the amide group and the carbonyl oxygen atom of an adjacent one (distances N1H1···O1’ 1.932(2) Å and N1···O1’ 2.877(2) Å; angle NHO 162.7(1)º). These dimers interact by double intermolecular F–F contacts between the F5 of a molecule and the F6 of a neighboring one (distance 2.875(2) Å) giving rise to a *zigzag* chain in the [10-1] direction (Figure 4). These chains are stacked by a partial π–π overlapping between the aromatic rings with a shortest distance of 3.19(1) Å.

**Figure 5:**
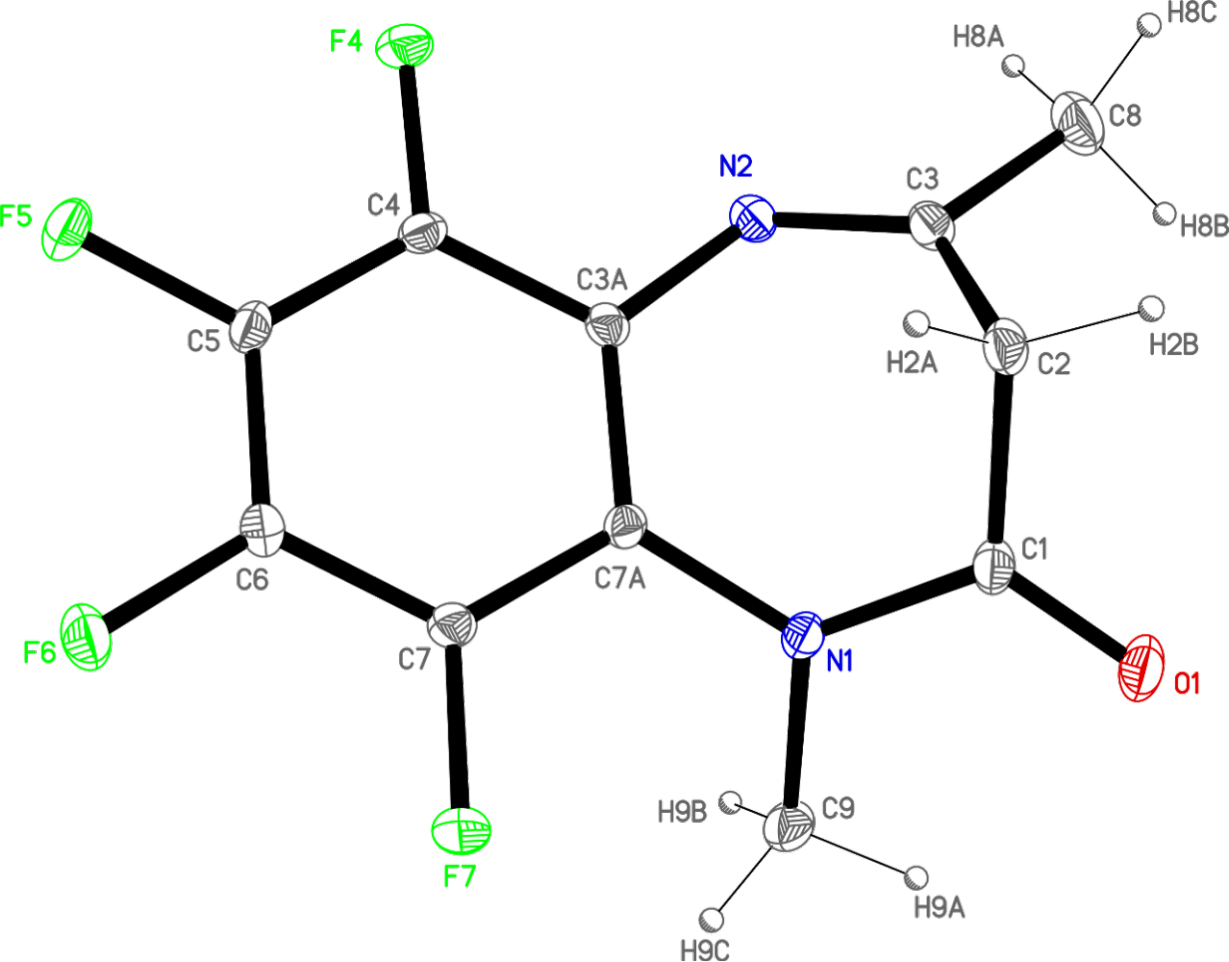
ORTEP plot (20% probability) of **2**, showing the X-ray labeling of the asymmetric unit.

Compound **2** crystallizes in the triclinic *P*–1 space group containing one molecule per asymmetric unit (Figure 5). As for compound **1**, the molecular geometry corresponds to tautomer **a**. The seven-membered ring is also folded with dihedral angles between the aromatic ring and C1N1O1 of 44.1(3)º and with N2C3C8 of 43.2(3)º. This higher values compared to compound **1** indicate a greater deformation in the seven-membered ring owing to the presence of the *N*-methyl substituent.

**Figure 6:**
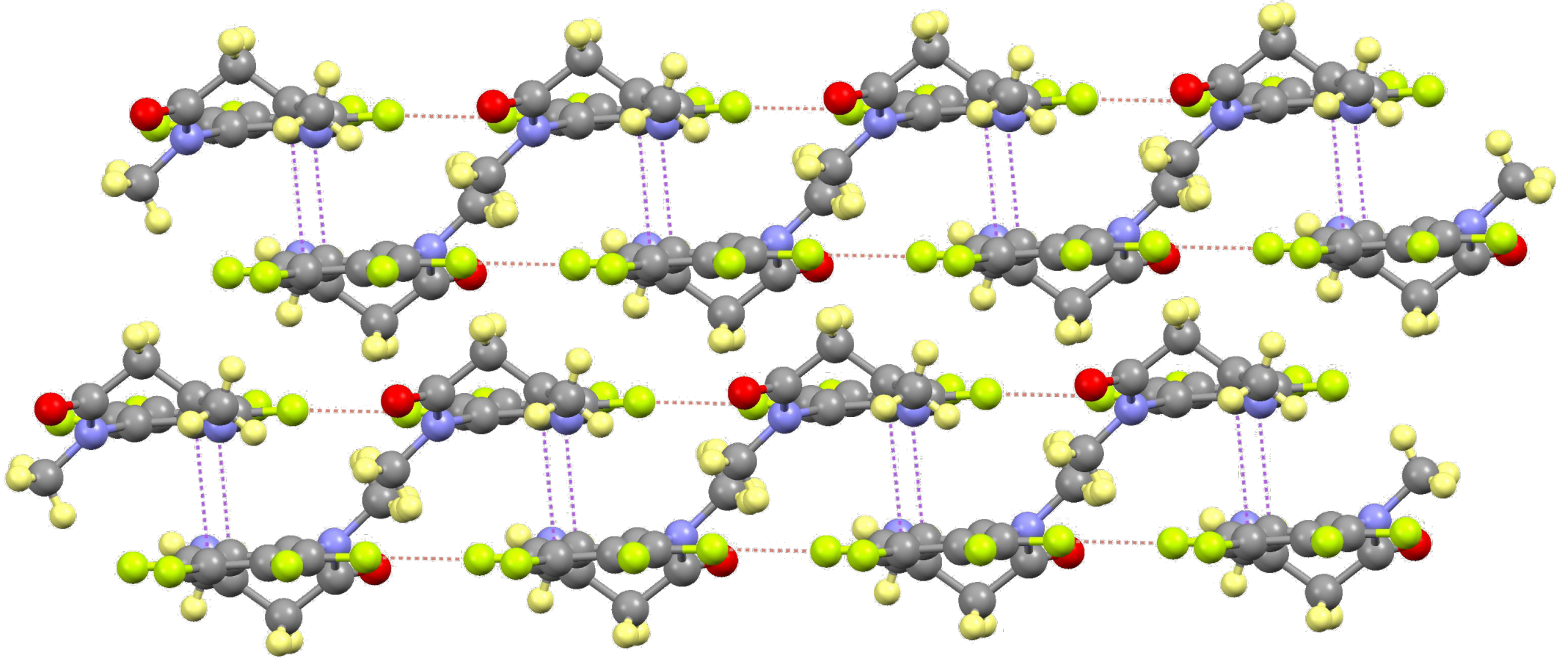
Packing of **2** showing the F–F contacts along the chain (orange) and the π–π interactions that form the double chain (violet).

The *N*-methylation prevents the dimerization by hydrogen bonding leading to a very different packing. Therefore, the most significant intermolecular interaction is the F–F contact between the F4 and F7 atoms of adjacent molecules (distance 2.669(3) Å) giving rise to chains along the *b* axis (Figure 6). Each chain is placed antiparallel to the following one in order to minimize the steric hindrance of the groups out of plane. The two chains interact by π–π overlapping between their aromatic rings, with a shortest distance of 3.35(1) Å. The so formed double chains are isolated because of the above-mentioned steric reasons.

We compared the geometries of compounds **3** (TUPSAZ) [8,10] and **5** (EFARUA) [8–10] (Figure 2) with those determined in the present work, **1** and **2**. To describe the folding of the seven-membered ring we used the distance *d* in Å between the methylene carbon and the plane defined by the benzene ring. These values are 1.36 Å (**3**), 1.26 Å (**5**), 1.33 Å (**1**) and 1.57 Å (**2**), thus the 1,5-benzodiazepinone with a 4-methyl ring, **3**, is more bent than that with a 4-phenyl ring, **5**. More significant for the present work, the *N*-methyl substituent folded considerably the ring, compare **2** with **1**, this being related to the inversion process discussed below.

### Energies and tautomerism

**Figure 7:**
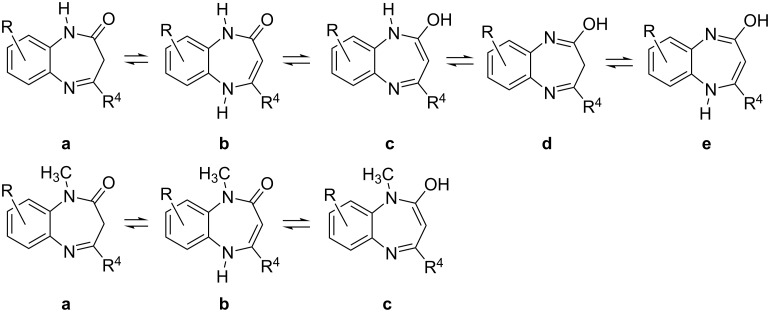
The different tautomers in the 1*H* and 1-methyl series.

For the 1*H*-derivatives five possible tautomers exist while for the *N*-methyl ones only three different tautomers are possible (Figure 7).

**Figure 8:**
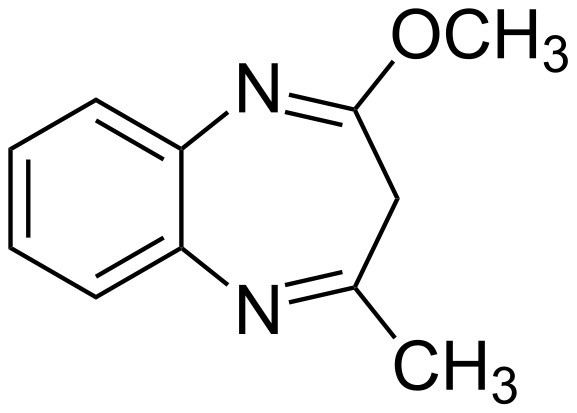
2-Methoxy-4-methyl-3*H*-1,5-benzodiazepine (**7**).

Mannschreck et al. already concluded in 1967 that **3** has the structure **3a** based on a methylene signal at 3.14 ppm [11]. This is also compatible with tautomer **3d** but considering that amides never exist as imidic acids, Mannschreck's conclusion is certainly right. Varma et al. reported in 2008 that the reaction between *o*-phenylenediamine and methyl acetylacetate yields the methoxy derivative **7** (Figure 8) without any reported proof [12]. In a subsequent paper they reported that the reaction of *o*-phenylenediamine using ethyl acetylacetate instead of methyl acetylacetate yielded the expected diazepinone that they represent using the tautomer **3d** again without any reported proof, neither in the main text nor in the supplementary data [13].

A comprehensive theoretical study of the tautomerism of **3** was carried out by Okovytyy et al. in 2010, including monoethanol and diethanol solvates as well as dimeric forms [14] (they do not consider tautomer **3e**). In the gas phase their relative energies (in kJ mol^−1^) are: **3a** (0.0) > **3b** (14.6) > **3d** (48.5) > **3c** (93.4) and with an ethanol molecule they are: **3a** (0.0) > **3b** (0.6) > **3d** (51.3) > **3c** (70.0). The great stabilization of **3b** due to ethanol does not correspond to that observed by Mannschreck in CDCl_3_ [11]. Our calculations (gas phase) are reported in Table 1.

**Table 1:** Relative stabilities in kJ mol^–1^ of the different tautomers of compounds shown in Figure 1 and Figure 7.

Compound	**a**	**b**	**c**	**d**	**e**

**1**	0.0	9.0	90.0	45.8	55.0
**2**	0.0	18.6	99.7	–	–
**3**	0.0	14.7	96.5	48.6	64.0
**4**	0.0	22.5	105.7	–	–
**5**	0.0	16.6	96.6	48.4	66.2
**6**	0.0	24.4	105.1	–	–

Our results agree with those of Okovytyy et al. [14] now including **3e**: **3a** (0.0) > **3b** (14.7) > **3d** (48.6) > **3e** (64.0) > **3c** (96.5). For the remaining compounds always is **a** (0.0) > **b** (18 kJ mol^−1^ in average), the other tautomers having considerably higher energies. Always tautomer **b** is destabilized by *N*-methylation (in average, 8.4 kJ mol^−1^) probably due to a steric effect; the conjugated tautomer **b** tends to be planar and this is indeed the case for 1*H*-derivatives **1b**, **3b** and **4b**. The introduction of an *N*-methyl group, derivatives **2b**, **4b** and **6b**, fold the seven-membered ring with a concomitant destabilization of these tautomers.

The tautomerism between **a** and **b** implies the breaking/formation of a C–H bond. This is similar to the case of acetylacetone (diketo and ketoenol tautomers) that when both tautomers are present, both can be observed by NMR because the tautomerization barrier is high enough. Therefore, if a CH_2_ group is observed in ^1^H or in ^13^C NMR in the case of 1,5-benzodiazepinones only tautomer **a** is present in solution.

### Chemical shifts and spin–spin coupling constants (SSCC)

Mannschreck et al. reported the ^1^H NMR chemical shifts (δ in ppm) of **3a** in CDCl_3_: 2.38 (CH_3_), 3.14 (CH_2_) and 9.99 (NH) [11]. Benasi et al. reported those of **5a** (δ_A_ = 3.08, δ_B_ = 4.25, *J*_AB_ = 12.00 Hz) and **6a** (δ_A_ = 3.02, δ_B_ = 4.15, *J*_AB_ = 12.00 Hz; CH_3_, 3.34) in acetone [15]. Those of **5a** in DMSO-*d*_6_ are 3.87 and 4.62 ppm [16]. A paper by Bernardini et al. reports all the ^13^C NMR chemical shifts and some ^1^H–^13^C coupling constants for compounds **3**–**6** [17].

We report in Table 2 (^1^H and ^19^F NMR data) and Table 3 (^13^C and ^15^N NMR data) the results we have obtained for compounds **1** and **2** in different solvents and in the solid state together with the theoretical calculated values.

**Table 2:** ^1^H and ^19^F chemical shifts (δ, ppm, Δδ = δ_eq_ − δ_ax_) and SSCC (^1^H–^1^H; ^1^H–^19^F; ^19^F–^19^F Hz) of compounds **1** and **2** at 300 K together with the calculated values in ppm and Hz.

Comp.	Conditions	CH_3_	CH_2_	NR	F6	F7	F8	F9

**1**	CDCl_3_	2.46	3.24 Δδ = 0.00	R = H 8.08	−147.8 ^3^*J*_F7_ = 21.6 ^5^*J*_F9_ = 9.6	−161.9 ^3^*J*_F8_ = 21.6	−159.7 ^3^*J*_F9_ = 21.6	−154.5
**1**	toluene-*d*_8_	1.83	2.39 Δδ = 0.00	R = H 7.80	−148.5 ^3^*J*_F7_ = 21.7 ^5^*J*_F9_ = 9.6	−163.8 ^3^*J*_F8_ = 21.7	−161.7 ^3^*J*_F9_ = 21.7	−154.8
**1**	DMSO-*d*_6_	2.32	3.24 Δδ = 0.00	R = H 10.66	−149.8 ^3^*J*_F7_ = 22.7 ^5^*J*_F9_ = 7.5	−164.6 ^3^*J*_F8_ = 22.7	−162.5 ^3^*J*_F9_ = 22.7	−151.1
**1**	solid state^a^	–	–	–	−144.9	−161.5	−159.0	−150.6
**1**	calculated	2.31	2.49 (ax) 3.23 (eq) Δδ = 0.74 *J*_ae_ = −10.6^b^	R = H 6.71	−142.8 ^3^*J*_F7_ = −22.0 ^4^*J*_F8_ = −1.5 ^5^*J*_F9_ = +11.6	−163.0 ^3^*J*_F8_ = −21.9 ^4^*J*_F9_ = −6.0	−160.0 ^3^*J*_F9_ = −22.0	−159.0 ^4^*J*_F7_ = −6.0

**2**	CDCl_3_	2.43	2.93 (ax) 3.53 (eq) Δδ = 0.60 *J*_ae_ = 11.7	R = Me 3.30 (d) ^5^*J*_F9_ = 4.2	−148.5 ^3^*J*_F7_ = 20.7 ^5^*J*_F9_ = 8.8	−159.7 ^3^*J*_F8_ = 20.7	−159.8 ^3^*J*_F9_ = 20.7	−145.2^b^
**2**	toluene-*d*_8_	1.85	2.03 (ax) 2.86 (eq) Δδ = 0.83 *J*_ae_ = 11.6	R = Me 2.88 (d) ^5^*J*_F9_ = 4.4	−148.7 ^3^*J*_F7_ = 21.4 ^5^*J*_F9_ = 8.4	−161.3 ^3^*J*_F8_ = 21.4	−161.6 ^3^*J*_F9_ = 21.4	−146.3 ^4^*J*_F7_ = 8.4 ^5^*J*_CH3_ = 4.4
**2**	DMSO-*d*_6_	2.31	3.19 (ax) 3.45 (eq) Δδ = 0.27 *J*_ae_ = 12.1	R = Me 3.18 (d) ^5^*J*_F9_ = 4.6	−150.0^c^	−162.1^c^	−162.1^c^	−144.7^c^
**2**	solid state^a^	–	–	–	−146.0	−156.8	−160.9	−142.1
**2**	calculated	2.27	2.47 (ax) 3.20 (eq) Δδ = 0.73 *J*_ae_ = −10.3	R = Me 3.13 (d) ^5^*J*_F9_ = 6.6	−144.3 ^3^*J*_F7_ = −22.2 ^4^*J*_F8_ = −2.8 ^5^*J*_F9_ = +10.6	−160.4 ^3^*J*_F8_ = −22.1 ^4^*J*_F9_ = −3.3	−160.7 ^3^*J*_F_ = −21.6	−146.1 ^4^*J*_F7_ = −3.3

^a^Obtained using the hpdec.av sequence; ^b^This coupling constant agrees with the experimental one found at 193 K in toluene-*d*_8_ (see Barriers section); ^c^Complex multiplet.

**Figure 9:**
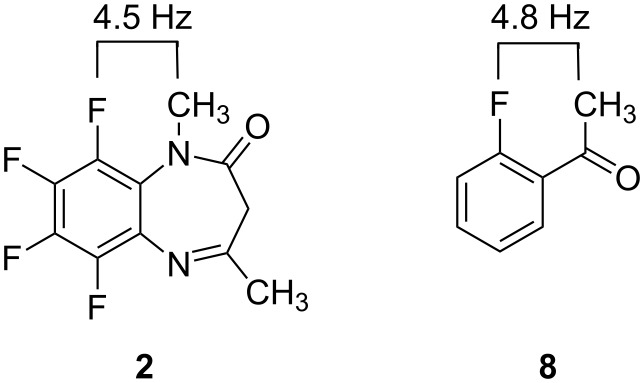
^1^H–^19^F coupling constant values either through-bond or through-space.

There are several interesting results concerning the data reported in Table 2. One of them is the *J*_HF_ coupling constant present in the *N*-methyl group of compound **2**. This coupling constant, of about 4.5 Hz, identifies unambiguously F9, i.e. it is a ^5^*J*_HF9_ because all the calculated values for the coupling constants between the *N*-methyl protons and the fluorine atoms are very small (about 0.1 Hz) except that with F9 (calculated 6.6 Hz). In the literature (Figure 9), there is a related coupling constant present in 2-fluoroacetophenone (**8**) [18]. Note that this ^1^H–^19^F coupling can be through-bonds, i.e. a ^5^*J* or through-space, a common problem involving ^19^F [19–20]. Starting from the F9 assignment, the (^19^F–^19^F) COSY experiments permitted to establish the correlation F9–F8–F7–F6, in both compounds.

Another interesting coupling is the geminal ^2^*J*_HH_ ≈ 12 Hz of the methylene group in the case of compound **2**. This coupling is well reproduced by the calculations ≈ −10.5 Hz. Next, we compared the experimental and calculated chemical shifts (Table 2). For ^1^H and ^19^F all together, (*n* = 44 values in solution) the correlations are very good (*R*^2^ = 1.000) but this is due to the fact that these nuclei appear at very different chemical shifts. Considering only the protons and not including the NH protons (*n* = 20), the regressions are good, for instance, δCDCl_3_ = −(0.4 ± 0.1) + (1.26 ± 0.04) δ_Calcd_ *R*^2^ = 0.995.

The part concerning ^19^F chemical shifts is less satisfactory. There are 32 values including those determined in the solid state. Imposing the intercept to be 0 (and considering that the calculations do not reproduce well the chemical shifts of F6 and F9, we found δ^19^F = (1.005 ± 0.003) δ_Calcd_ − (4.4 ± 0.7) F6 + (4.0 ± 0.7) F9 + (2.7 ± 0.7) CPMAS. The worse points are **1** DMSO-*d*_6_, F9 exp. −151.1, fitted −155.8; **2** toluene-*d*_8_, F9 exp. −146.3, fitted −142.9. The reason of this anomaly concerning the fluorine atoms closer to the nitrogen atoms remains unclear.

In what concerns the FF coupling constants, ^3^*J*_FF_ and ^5^*J*_FF_ agree with the calculated values; the average values being: experimental |21.4| (^3^*J*_F_) and |9.1| (^3^*J*_F9_) and calculated −22.0 (^3^*J*_F_) and 11.1 Hz (^3^*J*_F9_).

Table 3 reports the ^13^C and ^15^N NMR data; here the situation is more difficult because the ^13^C NMR signals of the benzene ring carbons are coupled with all the fluorine atoms giving rise to multiplets, which have been analyzed using the Mnova 8.1.0 NMR software [21] for spin simulation, and when needed by irradiation of the ^1^H nuclei to simplify the spectra. In the gs-HMBC (^1^H–^13^C) spectra, a correlation between the C4–CH_3_ protons and C4 permitted to assign to the latter the chemical shifts at 168.7 ppm for **1** and 170.9 for **2**, in accordance with the calculated values. However, the ^13^C CPMAS signals corresponding to carbon atoms C6 to C9 could not be properly analyzed, and only the centers of the multiplets are given (138.1 and 138.6 ppm for **1** and **2**, respectively). Some couplings involving the fluorine atoms are not well reproduced by the calculations, this is particularly apparent for the ^1^*J*_CF_, that are overestimated, in absolute value, by about 68 Hz. The overestimation and difficulty to calculate coupling constants involving ^19^F has been reported several times [20,22–23].

**Table 3:** ^13^C and ^15^N NMR chemical shifts (δ, ppm) and some coupling constants (Hz) of compounds **1** and **2** in solution and in the solid state together with the calculated values (ppm and Hz).

Comp.	Conditions	C2	C3	C4	4-CH_3_	*N*-CH_3_	C5a	C9a

**1**	DMSO-*d*_6_	165.3	44.6	168.7	27.6	–	126.6 ^2^*J* = 9.9	117.1 ^2^*J* = 13.1
**1**	CPMAS	167.1	44.4	169.2	25.6	–	125.2	116.5
**1**	*Calcd*	*161.4*	*44.5*	*165.8*	*27.9*	*–*	*126.5* *^2^J_F6 _= 4.0*	*116.2* *^2^J_F9 _= 3.4*
**2**	DMSO-*d*_6_	165.0	43.7	170.9	27.4	35.8 *^4^J*_F9_ = 8.7	128.4 ^2^*J* = 11.2	121.2 ^2^*J* = 8.9
**2**	CPMAS	165.8	42.6	173.3	27.3	36.2	128.7	121.5
**2**	*Calcd*	*162.3*	*44.1*	*168.9*	*27.4*	*36.9* *^4^J_F9 _= 8.5*	*129.8* *^2^J_F6 _= 2.7*	*122.2* *^2^J_F9 _= 2.1*

		C6	C7	C8	C9	N1	N5	

**1**	DMSO-*d*_6_	140.9 ^1^*J* = 246.0 ^2^*J* = 10.5 ^3^*J* = 3.7	136.3 ^1^*J* = 245.3 ^2^*J* ≈ 13.5 ^2^*J* ≈ 11.6 ^3^*J* = 3.8	137.3 ^1^*J* = 246.0 ^2^*J* ≈ 14.0 ^2^*J* ≈ 13.2 ^3^*J* = 4.4	138.2 ^1^*J* = 245.3 ^2^*J* = 11.8 ^3^*J* = 3.8	*−*254.3	*−*86.9	
**1**	CPMAS	138.1^a^	138.1^a^	138.1^a^	138.1^a^	–249.6	*−*85.0	
**1**	*Calcd*	*145.2* *^1^J = −316.6*	*139.8* *^1^J = −314.4*	*140.7* *^1^J = −316.7*	*139.1* *^1^J = −303.3*	*−256.7* ^3^*J_N1F9 _= −1.9*	*−82.0* ^4^*J_N5F7 _= 1.5* ^3^*J_N5F6 _= 5.2*	
**2**	DMSO-*d*_6_	140.6 ^1^*J* = 251.4 ^2^*J* = 12.1 ^3^*J* = 5.0	137.3 ^1^*J* = 248.3 ^2^*J* ≈ 16.2 ^2^*J* ≈ 15.4 ^3^*J* = 4.8	137.3	140.2 ^1^*J* = 241.8 ^2^*J* = 14.6 ^3^*J* = 4.9	*−*264.3	*−*90.5	
**2**	CPMAS	138.6^a^	138.6^a^	138.6^a^	138.6^a^	*−*259.2	*−*90.2	
**2**	*Calcd*	*143.8* *^1^J = −316.1*	*140.7* *^1^J = −315.5*	*140.3* *^1^J = −315.3*	*142.8* *^1^J = −310.3*	*−256.2* ^3^*J_N1F9 _= −2.1*	*−85.0* ^4^*J_N5F7 _= 1.4* ^3^*J_N5F6 _= 5.2*	

^a^Very complex multiplet.

Concerning the ^15^N experimental spectra all nitrogen atoms appear as singlets in solution as well as in the solid state, only small coupling constants with the fluorine substituents have been theoretically calculated so most probably the width of the experimental signals mask them.

In Table S1 in Supporting Information File 1 the experimental (from references [11,15–17]) and calculated ^1^H, ^13^C and ^15^N NMR chemical shifts (δ, ppm) of compounds **3a** to **6a** are reported. The agreement between experimental and calculated values is either good or excellent.

### Barriers (all in kJ mol^−1^)

The experimental inversion barriers of **3a**, **5a** (twice) and **6a** have been determined and are given in Table 4. We have calculated those of **1a** and **2a**. The barriers for an AB system that become an A_2_ one depend on three values: i) the coalescence temperature *T*_C_; ii) the difference in Hz of the protons of the AB system (Δ*ν*_AB_), and iii) the geminal coupling constant, *J*_AB_ (or *J*_ae_). The inversion rate at the coalescence temperature for an AB system is given by *k*_C_ = π/√2·√Δ*ν*^2^ + 6 *J*_AB_^2^ [11] and the barrier by the modified Eyring equation [24–26], Δ*G*^‡^ = 19.12·*T*_C_ (10.32 + log *T*_C_/*k*_C_) [18–20]. From the values in Table 4 we have determined the corresponding experimental inversion barriers.

**Table 4:** Experimental al calculated inversion barriers (kJ mol^–1^).

	**1a**	**2a**	**3a**	**4a**	**5a**	**6a**

Δ*G*^‡^_exp._	toluene-*d*_8_ *T*_C_ = 230 K Δν_AB_ = 450.0 Hz *J*_AB_ = 10.9 Hz 42.6	DMSO-*d_6_* *T*_C_ = 373 K Δν_AB_ = 103.5 Hz *J*_AB_ = 12.1 Hz 75.0 toluene-*d_8_* *T*_C_ = 363 K Δν_AB_ = 303.1 Hz *J*_AB_ = 11.6 Hz 69.8	pyridine-*d*_5_/CDCl_3_ 39.8 [11]	–	acetone-*d*_6_ 41.8 [15] 52.7 [16]	acetone-*d*_6_ 65.3 [15]
Δ*G*^‡^_calcd._	36.7	81.7	42.4	61.9	38.2	63.8

The agreement between experimental and calculated values is satisfactory: using for **2a** the 75.0 kJ mol^−1^ and for **5a** the 41.8 kJ mol^−1^ values we obtained by linear regression (no intercept) Δ*G*^‡^_exp._ = (0.99 ± 0.04) Δ*G*^‡^_calcd_, *n* = 5, *R*^2^ = 0.993. This equation predicts for **4a** 61.0 kJ mol^−1^. Note the increase between toluene and DMSO that corresponds to the raise of inversion barriers with that of the solvent polarity; a similar behavior has been reported for diazepam (7-chloro-1,3-dihydro-1-methyl-5-phenyl-2*H*-1,4-benzodiazepin-2-one) [27].

When using calculated values it is possible to analyze the main effects on the barriers that in the present case are three: i) *N*-methylation; ii) 6,7,8,9-tetrafluorination; iii) the substituent at position 4 (CH_3_ or C_6_H_5_). This last effect is negligible, the other two interact, then a product term (i · ii) is necessary to model the behavior. The regression corresponds to the following equation (all values in kJ mol^−1^):

Calculated barrier = (40.3 ± 1.6) + (22.6 ± 2.3) *N*Me − (3.6 ± 2.8) C_6_F_4_ + (22.5 ± 4.0) *N*Me*C_6_F_4_, *n* = 6, *R*^2^ = 0.993

**Figure 10:**
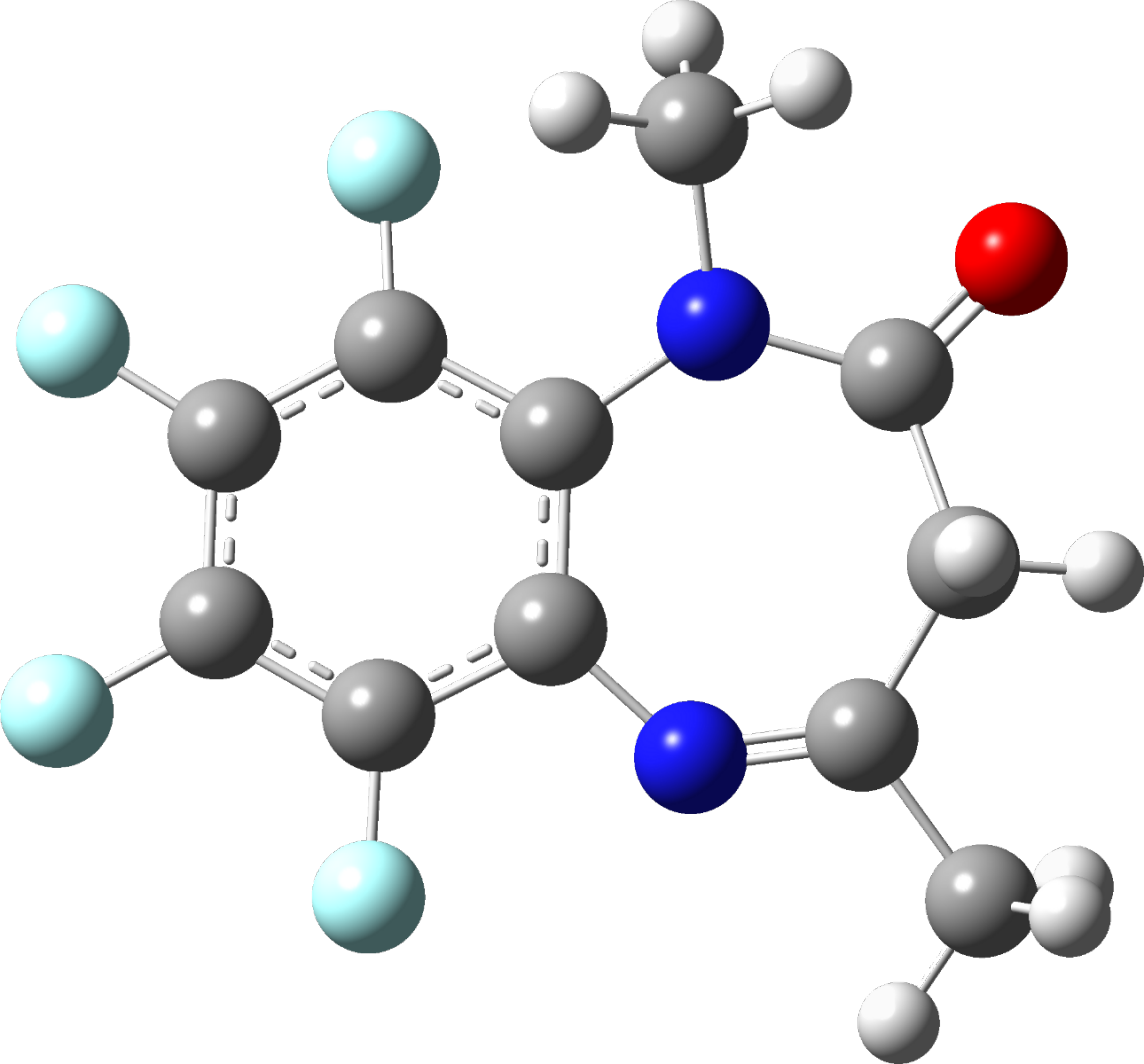
The optimized geometry of the TS of **2a** inversion.

The average barrier is 40.3 kJ mol^−1^. *N*-Methylation increases the barrier 22.6 kJ mol^−1^ in average; the introduction of four F atoms produces a small effect of 3.6 kJ mol^−1^ but when there is simultaneously an *N*-methyl group and four F atoms (compound **2a**), the increase of the barrier is considerable (22.5 kJ mol^−1^). An examination of the TS for **2a** shows that the protons of the *N*-methyl group are very close to F9 (Figure 10).

## Conclusion

A clearer picture of the behavior of 1,5-benzodiazepinones emerges from the present paper. Concerning tautomerism, our results confirm previous studies while extending them to the 6,7,8,9-tetrafluoro derivatives. The folding of the structures in the solid-state is general, the *N*-methyl group having a marked effect. An exhaustive NMR study of the tetrafluoro derivatives, together with literature data on four other non-fluorinated 1,5-benzodiazepinones, lead to values of the chemical shifts that compare well with GIAO calculated ones with the exception of F6 and F9. Finally, the inversion barrier of the seven-membered ring of the new compounds has been determined and compared with theoretically calculated values, illustrating the considerable effect of the *N*-methylation particularly when there is a fluorine atom on C9.

## Experimental

**General Information.** All chemicals cited in the synthetic procedures are commercial compounds. Melting points were determined by DSC with a SEIKO DSC220C connected to a model SSC5200H disk station. Thermograms (sample size 0.004 g) were recorded with a scan rate of 5.0 °C. Column chromatography was performed on silica gel (Merck 60, 70–230 mesh) and elemental analyses using a Perkin-Elmer 240 apparatus.

**6,7,8,9-Tetrafluoro-4-methyl-1,3-dihydro-2*H*-1,5-benzodiazepin-2-one (1)**. 1,2-Diamino-3,4,5,6-tetrafluorobenzene (0.50 g, 2.78 mmol) and ethyl acetylacetate (0.36 mL, 2.85 mmol) were heated at 120 °C in anhydrous xylene (5 mL) for 6 hours. The mixture was cooled and then a solid precipitated. This residue was washed with diethyl ether to give compound **1** (0.43 g, 63%); white solid; mp 155.2 °C. Anal. calcd for C_10_H_6_F_4_N_2_O: C, 48.79; H, 2.46; N, 11.38; found: C, 48.04; H, 2.60; N, 11.41.

**6,7,8,9-Tetrafluoro-1,4-dimethyl-1,3-dihydro-2*H*-1,5-benzodiazepin-2-one (2).** A solution of **1** (0.40 g, 1.62 mmol) in DMF (2 mL) was heated at 110 °C in the presence of iodomethane (0.11 mL, 1.79 mmol), K_2_CO_3_ (0.27 g, 1.95 mmol) and a catalytic quantity of KI for 90 min. The mixture was cooled, treated with cold water and extracted with ethyl acetate. The ethyl acetate was evaporated and the crude was purified by column chromatography (hexane/ethyl acetate 7:3) to afford compound **2** (0.34 g, 80%); pale yellow solid; mp 132.2 °C. Anal. calcd for C_11_H_8_F_4_N_2_O: C, 50.78; H, 3.10; N, 10.77; found: C, 50.91; H, 3.15; N, 10.76.

**X-ray data collection and structure refinement.** Data collection for all compounds was carried out at room temperature on a Bruker Smart CCD diffractometer using graphite-monochromated Mo Kα radiation (λ = 0.71073 Å) operating at 50 kV and 30 mA for **1** and **2**. In all cases, data were collected over a hemisphere of the reciprocal space by combination of three exposure sets. Each exposure was of 20 s covered 0.3 in ω. The cell parameter were determined and refined by a least-squares fit of all reflections. The first 100 frames were recollected at the end of the data collection to monitor crystal decay, and no appreciable decay was observed. A summary of the fundamental crystal and refinement data is given in Table 5.

**Table 5:** Crystal data and refinement data for **1** and **2**.

Crystal Data	**1**	**2**

Empirical formula	C_10_H_6_ F_4_N _2_O_1_	C_11_H_8_ F_4_N_2_O_1_
Formula wt	246.17	260.19
Crystal system. Space group	Monoclinic *P*2_1_/*c*	Triclinic *P*–1
*a*/Å	5.2821(5)	7.224(4)
*b*/Å	18.255(1)	8.064(4)
*c*/Å	10.2102(9)	10.478(5)
α/°		77.136(9)
β/°	100.93(1)	76.311(9)
γ/°		68.510(8)
*V*/Å^3^	966.6(1)	545.5(5)
*Z*	4	2
*D*_c_ /g/cm^3^	1.692	1.584
µ(Mo Kα) /mm^–1^	0.164	0.150
F(000)	496	264
*θ* range/°	2.23 to 25.01	2.02 to 25.00
Index ranges	−6, −18, −8 to 6, 21, 12	−8, −9, −12 to 7, 9, 12
Reflections collected	4110	4184
Unique reflections [*R*int]	1709 [*R*int = 0.0233]	1869 [*R*(int) = 0.0350]
Completeness to theta	100%	97.0%
Data/restraints/params	1709/0/155	1869/0/163
Goodness-of-fit on F^2^	1.006	0.999
*R*1 (reflns obsd) [I > 2*σ*(I)] ^a^	0.0386 (1267)	0.0443 (1306)
*wR*2 (all data) ^b^	0.0935	0.1539

^a^*R*1 = Σ||F_o_|− |F_c_||/Σ|F_o_|. ^b^*wR*2 = {Σ[*w*(F_o_^2^ − F_c_^2^)^2^]/Σ[*w*(F_o_^2^)^2^]}.

The structures were solved by direct methods and refined by full-matrix least-square procedures on F^2^ (SHELXL-97) [28]. All non-hydrogen atoms were refined anisotropically.

The hydrogen atoms were included in their calculated positions and refined riding on the respective carbon atoms with the exception of hydrogen H1 bonded to N1 for **1** that was located in a Fourier synthesis and refined riding on the respective bonded atom.

Further crystallographic details for the structure reported in this paper may be obtained from The Cambridge Crystallographic Data Center, on quoting the depository numbers CCDC 946878 and 946879. Copies of the data can be obtained free of charge on application to The Director, CCDC, 12 Union Road, Cambridge DB2 1EZ, UK (Fax: int. code +(1223)336-033; email: deposit@ccdc.cam.ac.uk).

**Theoretical calculations.** The geometry of the molecules has been fully optimized with the hybrid HF/DFT B3LYP [29–31] computational method and the 6-31G(d) basis set [32]. Frequency calculations have been carried out at the same computational level to verify that the structures obtained correspond to energetic minima. A further optimization has been carried out at the B3LYP/6-311++G(d,p) level [33–34]. These geometries have been used for the calculations of the absolute chemical shieldings with the GIAO method [35–36] and the B3LYP/6-311++G(d,p) computational level. All the calculations have been carried out with the Gaussian-09 package [37].

**Figure 11:**
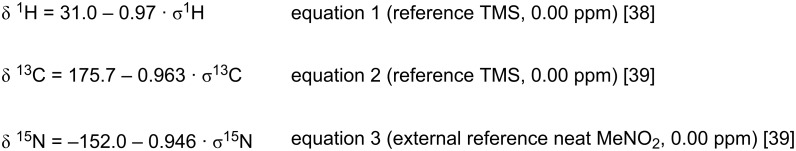
Equations used to transform absolute shieldings into chemical shifts [38–39].

The literature equations shown in Figure 11 have been used to transform absolute shieldings into chemical shifts.

### Experimental NMR

Solution spectra were recorded on a Bruker DRX 400 (9.4 Tesla, 400.13 MHz for ^1^H, 100.62 MHz for ^13^C, 40.56 MHz for ^15^N and 379.50 MHz for ^19^F) using a 5 mm QNP direct-detection probehead equipped with a z-gradient coil, at 300 K. Chemical shifts (δ in ppm) are given from internal solvent, DMSO-*d*_6_ 2.49 for ^1^H and 39.5 for ^13^C; CDCl_3_ 7.26 for ^1^H and toluene-*d*_8_ 2.09 for ^1^H, and for ^15^N and ^19^F NMR, nitromethane (0.00) and one drop of CFCl_3_ in CDCl_3_ (0.00) were used as external references.

Typical parameters for ^1^H NMR spectra were spectral width of 4800 Hz and pulse width of 10.25 μs at an attenuation level of –3.0 dB. Typical parameters for ^13^C NMR spectra were spectral width of 21 kHz, pulse width of 8.75 μs at an attenuation level of –3 dB and relaxation delay of 2 s, WALTZ-16 used for broadband proton decoupling; the FIDS were multiplied by an exponential weighting (lb = 1 Hz) before Fourier transformation. Typical parameters for ^19^F NMR spectra were spectral width of 55 kHz, pulse width of 13.75 μs at an attenuation level of –6 dB and relaxation delay of 1 s. WALTZ-16 was used for broadband proton decoupling ^19^F{^1^H}, the FIDS were multiplied by an exponential weighting (lb = 1 Hz) before Fourier transformation. Homonuclear ^19^F−^19^F experiment does not require any modification of the standard gs-COSY pulse sequences; selected parameters for ^19^F COSY spectra were: spectral width of 55 KHz, TD1 = 512 for *F*_1_ domain, spectral width of 55 KHz, TD1 = 1024 for *F*_2_ domain, number of scans 4, dummy scans DS = 2 and relaxation delay of 1 s.

Inverse proton detected heteronuclear shift correlation spectra, (^1^H–^13^C) gs-HMBC, were acquired and processed using standard Bruker NMR software and in nonphase-sensitive mode. Gradient selection was achieved through a 5% sine truncated shaped pulse gradient of 1 ms.

Inverse proton-detected heteronuclear shift correlation spectra, (^1^H–^15^N) gs-HMQC and (^1^H–^15^N) gs-HMBC, were acquired and processed using standard Bruker NMR software and in nonphase-sensitive mode. Gradient selection was achieved through a 5% sine truncated shaped pulse gradient of 1 ms. Selected parameters for (^1^H–^15^N) gs-HMQC and (^1^H–^15^N) gs-HMBC spectra were spectral width of 3500 Hz for ^1^H and 12.5 kHz for ^15^N, 1024 x 256 data set, number of scans 4, relaxation delay of 1 s, 37–60 ms delay for evolution of the ^15^N–^1^H long-range coupling. The FIDs were processed using zero filling in the *F_1_* domain and a sine-bell window function in both dimensions was applied prior to Fourier transformation.

Variable temperature was performed using a Bruker BVT3000 temperature unit to control the temperature of the cooling gas stream and an exchanger to achieve low temperatures. To avoid problems at low temperatures caused by air moisture, pure nitrogen was used as bearing, driving and cooling gas.

Solid state ^13^C (100.73 MHz) and ^15^N (40.60 MHz) CPMAS NMR spectra have been obtained on a Bruker WB 400 spectrometer at 300 K using a 4 mm DVT probehead. Samples were carefully packed in 4 mm diameter cylindrical zirconia rotors with Kel-F end-caps. Operating conditions involved 2.9 µs 90° ^1^H pulses and decoupling field strength of 86.2 kHz by TPPM sequence. ^13^C NMR spectra were originally referenced to a glycine sample and then the chemical shifts were recalculated to the Me_4_Si (for the carbonyl atom δ (glycine) = 176.1 ppm) and ^15^N NMR spectra to ^15^NH_4_Cl and then converted to nitromethane scale using the relationship: δ ^15^N(nitromethane) = δ ^15^N(ammonium chloride) − 338.1 ppm.

The typical acquisition parameters for ^13^C CPMAS were: spectral width, 40 kHz; recycle delay, 5 s; acquisition time, 30 ms; contact time, 2 ms; and spin rate, 12 kHz. In order to distinguish protonated and unprotonated carbon atoms, the NQS (non-quaternary suppression) experiment by conventional cross-polarization was recorded; before the acquisition the decoupler is switched off for a very short time of 25 μs [40–42]. And for ^15^N CPMAS were: spectral width, 40 kHz; recycle delay, 5 s; acquisition time, 35 ms; contact time, 6 ms; and spin rate, 6 kHz.

Solid-state ^19^F (376.94 MHz) NMR spectra have been obtained on a Bruker WB 400 spectrometer using a MAS DVT BL2.5 X/F/H trible resonance probehead. Samples were carefully packed in 2.5 mm diameter cylindrical zirconia rotors with Kel-F end-caps. Samples were spun at the magic angle at rates of 25 kHz and the experiments were carried out at ambient probe temperature.

Typical parameters for single pulse ^19^F MAS NMR spectra were: spectral width, 75 KHz; pulse width, 2.5 μs; recycle delay, 10 s; scans, 128; and spin rate, 25 kHz.

The typical acquisition parameters 19F{1H} MAS were: spectral width, 75 kHz; recycle delay, 10 s; pulse width, 2.5 μs and proton decoupling field strength of 100 kHz by SPINAL-64 sequence; recycle delay, 10 s; acquisition time, 25 ms; 128 scans; and spin rate, 25 kHz.

The ^19^F spectra were referenced to ammonium trifluoroacetate sample and then the chemical shifts were recalculated to the CFCl_3_ [δ (CF_3_COONH_4_^+^)] = –72.0 ppm)

## Supporting Information

Variable temperature ^1^H NMR spectra, ^13^C, ^15^N, ^19^F solid state NMR spectra; Table S1 containing calculated and some experimental ^1^H, ^13^C and ^15^N chemical shifts (δ, ppm) of compounds **3a** to **6a**; Geometry (Å), energy (hartree) and number of imaginary frequencies of the different tautomers calculated at the B3LYP/6-311++G(d,p) computational level.

File 1 Additional material.
