# Supplementary material for: Structure of 1,5-benzodiazepinones in the solid state and in solution: Effect of the fluorination in the six-membered ring
Source: Beilstein J Org Chem. 2013 Oct 21;9:2156–67. doi: 10.3762/bjoc.9.253 (PMC3817505; doi:10.3762/bjoc.9.253)
Supplement: File 1 — Additional material. [file Beilstein_J_Org_Chem-09-2156-s001.pdf]

Supporting Information

for

Structure of 1,5-benzodiazepinones in the solid state and in solution: Effect of the fluorination in the six-membered ring

Marta Pérez-Torralba[1], Rosa M. Claramunt*[1], Mª Ángeles García[1], Concepción López[1], M. Carmen Torralba[2], M. Rosario Torres[2], Ibon Alkorta[3] and José Elguero[3]

Address: [1]Departamento de Química Orgánica y Bio-Orgánica, Facultad de Ciencias, UNED, Paseo Senda del Rey 9, 28040-Madrid, Spain, [2]Departamento de Química Inorgánica I and CAI de Difracción de Rayos-X, Facultad de Ciencias Químicas, UCM, 28040-Madrid, Spain and [3]Instituto de Química Médica, Centro de Química Orgánica Manuel Lora-Tamayo, IQM-CSIC, Juan de la Cierva 3, 28006-Madrid, Spain

Email: Rosa M. Claramunt - rclaramunt@ccia.uned.es

*Corresponding author

Additional material

Figure S1: ^{1}H NMR spectra at several temperatures of compound **1** in toluene-d_8.

Figure S2: ^{1}H NMR spectra at several temperatures of compound **2** in toluene-d_8.

Figure S3: ^{13}C NMR spectra (CPMAS and NQS sequence) of compound **1**.

Figure S4: ^{15}N CPMAS NMR spectrum of compound **1**.

Figure S5: ^{19}F MAS NMR spectrum of compound **1**.

Figure S6: ^{13}C NMR spectra (CPMAS and NQS sequence) of compound **2**.

Figure S7: ^{15}N CPMAS NMR spectrum of compound **2**.

Figure S8: ^{19}F MAS NMR spectrum of compound **2**.

Table S1: Calculated and some experimental ^{1}H, ^{13}C and ^{15}N chemical shifts (δ, ppm) of compounds **3a** to **6a.**

Geometry (Å), energy (hartree) and number of imaginary frequencies of the different tautomers calculated at the B3LYP/6-311++G(d,p) computational level.

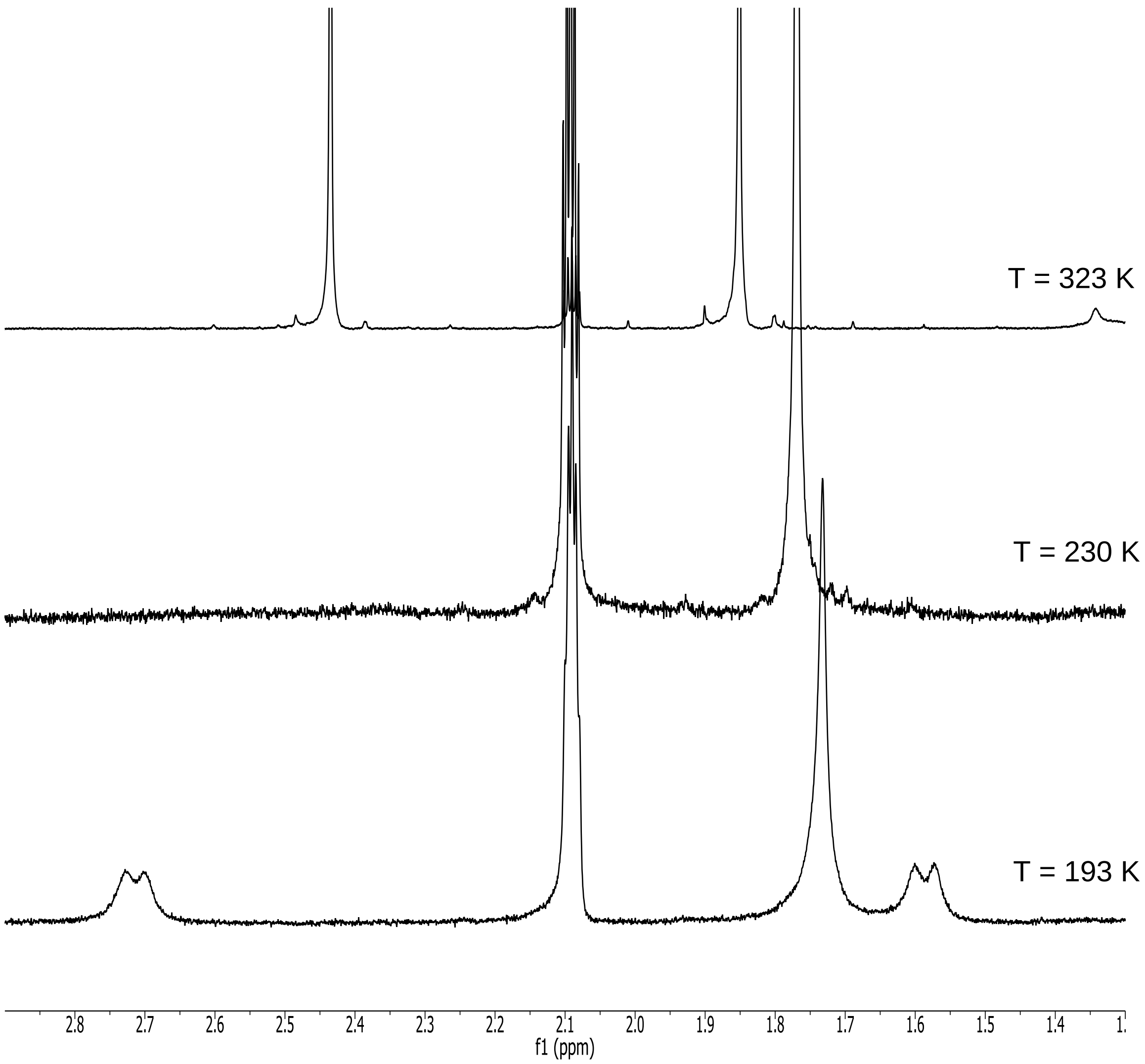

Figure S1: ^{1}H NMR spectra at several temperatures of compound **1** in toluene-d_8.

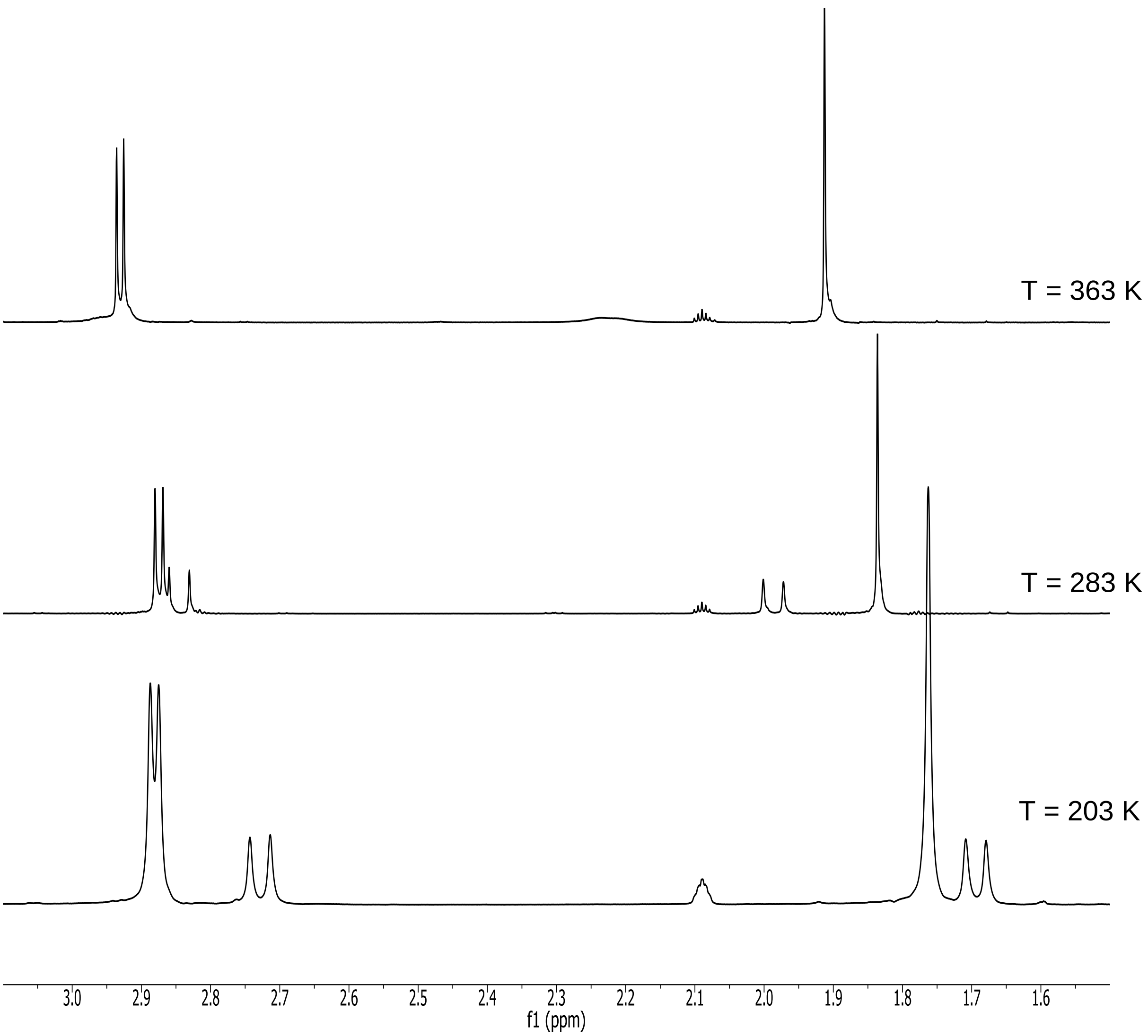

Figure S2: ^{1}H NMR spectra at several temperatures of compound **2** in toluene-d_8.

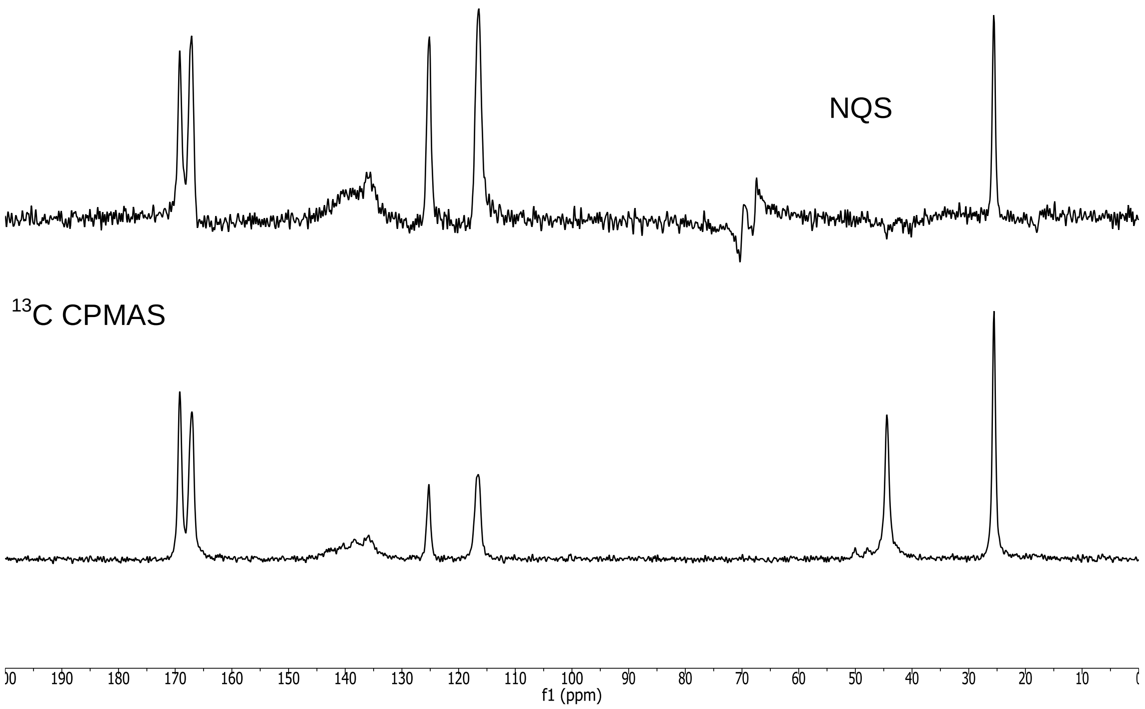

Figure S3: ^{13}C NMR spectra (CPMAS and NQS sequence) of compound **1**.

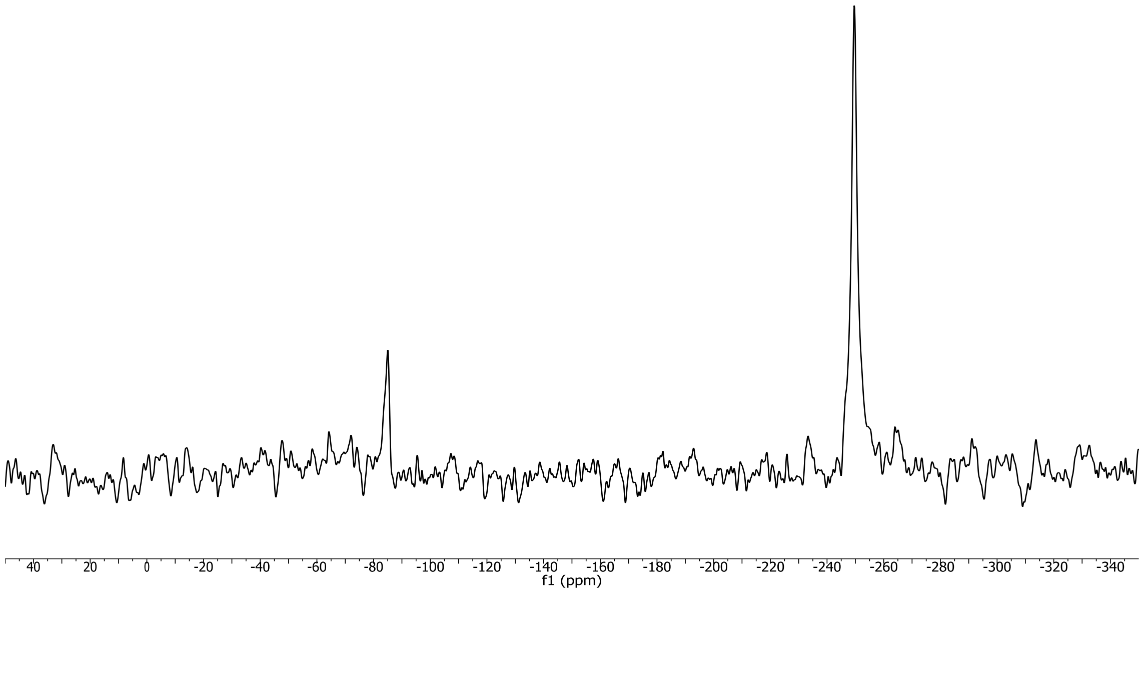

Figure S4: ^{15}N CPMAS NMR spectrum of compound **1**.

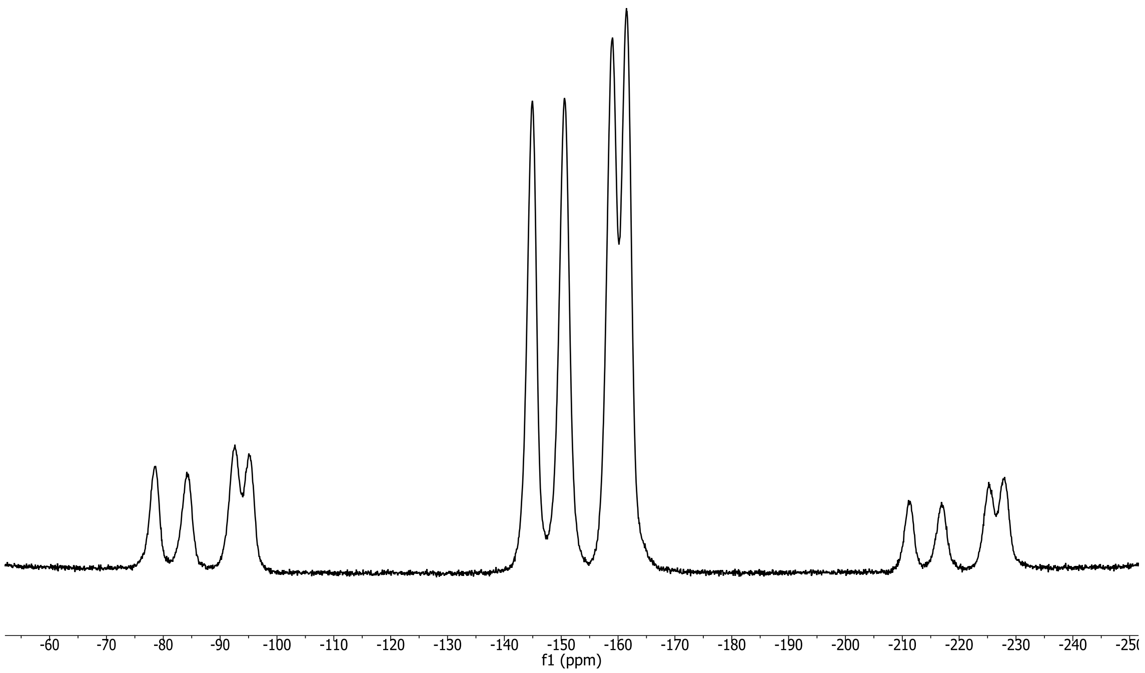

Figure S5: ^{19}F MAS NMR spectrum of compound **1**.

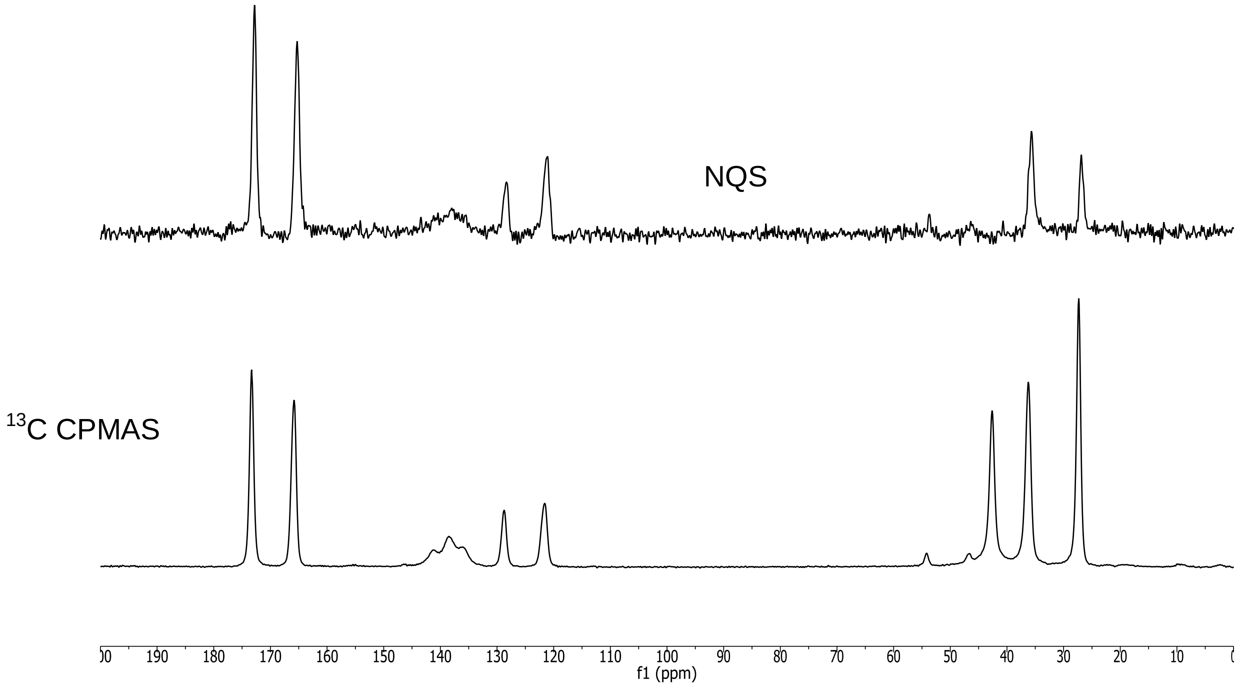

Figure S6: ^{13}C NMR spectra (CPMAS and NQS sequence) of compound **2**.

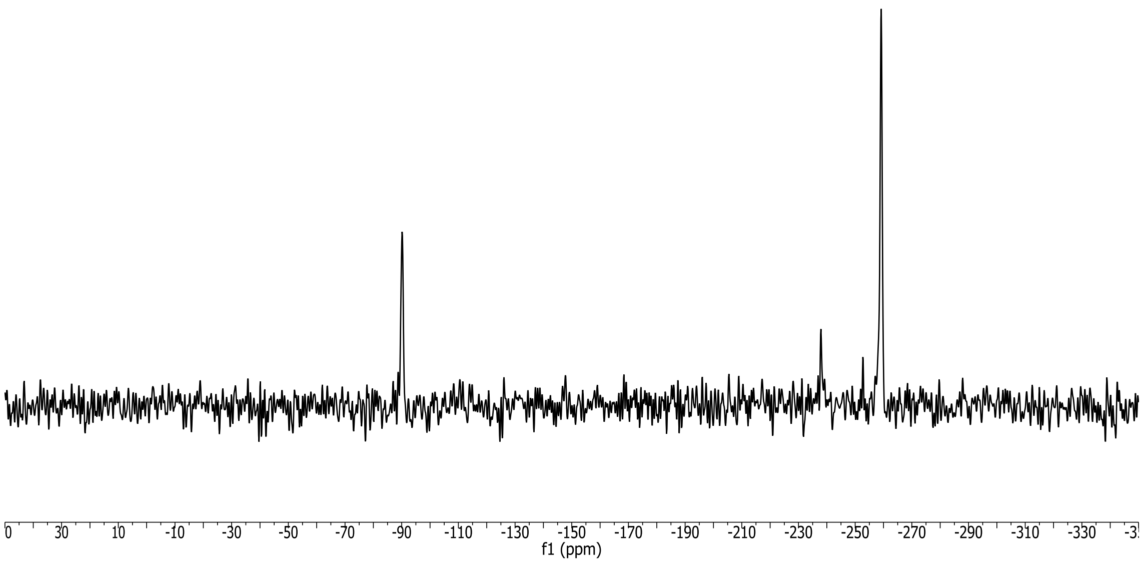

Figure S7: ^{15}N CPMAS NMR spectrum of compound **2**.

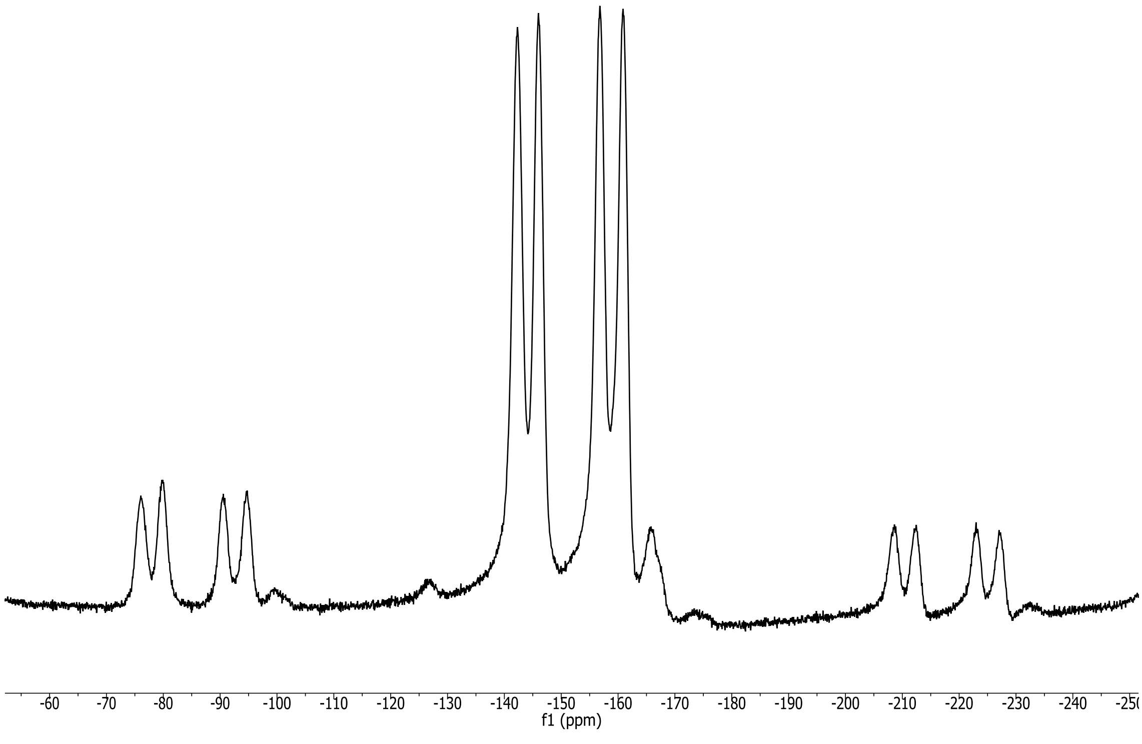

Figure S8: ^{19}F MAS NMR spectrum of compound **2**.

Table S1: Calculated and some experimental ^{1}H, ^{13}C and ^{15}N chemical shifts (δ, ppm) of compounds **3a** to **6a.**

Comp.	Nucleus	Solvent	NH	CH_2	4-CH_3	H6	H7	H8	H9
**3a**	^{1}H	(gas)	6.62	2.50 (ax)<br>3.12 (eq)	2.28<br>(CH_3)	7.40	7.10	7.05	6.78
Exp. [1]		$CDCl_3$	9.99	3.14 (s)	2.38	----	----	----	----
Exp. [2]		Acetone	----	3.08<br>4.25	----	----	----	----	----
Exp. [3]		DMSO-d_6	----	3.87<br>3.42	----	----	----	----	----
	^{13}C	(gas)	NH	C2	C3	C4	C5a	C6	C7
			----	162.8	43.4	162.4	141.2	129.3	123.3
			C8	C9	C9a	4-CH_3	N1	N5	
	^{13}C & ^{15}N		125.0	119.6	130.1	28.1	–245.0	–64.4	
Exp. [4]	^{13}C	$CDCl_3$	NH	C2	C3	C4	C5a	C6	C7
			----	167.6	43.7	163.1	140.0	127.9	125.2
	^{13}C & ^{15}N		C8	C9	C9a	4-CH_3	N1	N5	
			126.4	122.1	129.4	27.9	----	----16	
**4a**	^{1}H	(gas)	NCH_3	CH_2	4-CH_3	H6	H7	H8	H9
			3.25	2.49 (ax)<br>3.14 (eq)	2.23	7.34	7.08	7.13	7.14
	^{13}C	(gas)	NCH_3	C2	C3	C4	C5a	C6	C7
			34.2	163.6	43.7	164.0	143.1	128.1	123.4
	^{13}C & ^{15}N	(gas)	C8	C9	C9a	4-CH_3	N1	N5	
			124.4	120.0	137.0	27.5	–248.8	–66.8	
Exp. [4]	^{13}C	$CDCl_3$	NCH_3	C2	C3	C4	C5a	C6	C7

			40.0	165.7	43.5	165.0	141.4	126.4	125.1
	^{13}C & ^{15}N		C8	C9	C9a	4-CH_3	N1	N5	
			125.7	121.9	135.3	27.4	----	----	
**5a**	^{1}H	(gas)	NH	CH_2	4-C_6H_5	H6	H7	H8	H9
			6.73	2.58 (ax)<br>3.82 (eq)	8.30 (*o*)<br>7.44 (*m*)<br>7.40 (*p*)	7.47	7.14	7.12	6.85
	^{13}C	(gas)	NH	C2	C3	C4	C5a	C6	C7
			----	163.8	40.2	158.2	141.3	129.3	123.6
			C8	C9	C9a	4-C_6H_5	N1	N5	
			125.1	119.7	130.6	139.0 (*i*)<br>128.4 (*o*)<br>127.9 (*m*)<br>130.4 (*p*)	–243.5	–67.7	
Exp. [4]	^{13}C	$CDCl_3$	NH	C2	C3	C4	C5a	C6	C7
			----	167.8	39.8	158.6	139.9	128.3	126.1
	^{13}C & ^{15}N		C8	C9	C9a	4-C_6H_5	N1	N5	
			126.5	121.8	129.1	137.6 (*i*)<br>128.7 (*o*)<br>127.7 (*m*)<br>130.9 (*p*)			
**6a**	^{1}H	(gas)	NCH_3	CH_2	4-C_6H_5	H6	H7	H8	H9
			3.27	2.63 (ax)<br>3.86 (eq)	8.33 (*o*)<br>7.42 (*m*)<br>7.40 (*p*)	7.43	7.18	7.19	7.13
	^{13}C	(gas)	NCH_3	C2	C3	C4	C5a	C6	C7

			34.3	164.6	40.4	159.8	143.0	128.1	123.6
	^{13}C & ^{15}N		C8	C9	C9a	4-C_6H_5	N1	N5	
			124.4	120.2	137.7	137.8 (*i*)<br>128.1 (*o*)<br>127.9 (*m*)<br>130.3 (*p*)			
Exp. [4]	^{13}C	$CDCl_3$	NCH_3	C2	C3	C4	C5a	C6	C7
			35.0	166.4	39.7	160.9	141.9	127.5	125.2
	^{13}C & ^{15}N		C8	C9	C9a	4-C_6H_5	N1	N5	
			126.2	121.8	135.5	137.7 (*i*)<br>128.8 (*o*)<br>127.9 (*m*)<br>131.0 (*p*)	----	----	

References:

1. Mannschreck, A.; Rissmann, G.; Vögtle, F.; Wild, D. *Chem. Ber*. **1967**, *100*, 335–346. doi:0.1002/cber.19671000138

2. Benasi, R.; Lazzeretti, P.; Taddei, F.; Nardi, D.; Tajana, A. *Org. Magn. Reson*. **1976**, *8*, 387–388. doi:10.1002/mrc.1270080715

3. Chidichimo, G.; Longeri, M.; Menniti, G.; Romeo, G.; Ferlazzo, A. *Org. Magn. Reson*. **1984**, *22*, 52–54. doi:10.1002/mrc.1270220111

4. Bernardini, A.; Viallefont, P.; Essassi, E. M.; Zniber, R. *Org. Magn. Reson*. **1982**, *18*, 134–137. doi:10.1002/mrc.1270180304

Geometry (Å), energy (hartree) and number of imaginary frequencies of the different tautomers calculated at the B3LYP/6-311++G(d,p) computational level.

01_a, Total Energy= -969.025490094 Hartree, NIMAG= 0
C,-0.3194797381,0.2228050892,-0.6726661843
C,-0.2098346491,0.2019524789,0.7389418464
C,0.153030365,1.3983610587,1.3846392312
C,0.3812150847,2.5728952831,0.6911514029
C,0.2357135536,2.5924589382,-0.6949006825
C,-0.1070383204,1.42773057,-1.3539169658
N,-0.5707146419,-0.8707968744,1.5474680733
N,-0.7117165322,-0.8897409151,-1.4264625248
C,-0.3356722304,-2.2106851678,-1.2422233566
C,-0.3060667168,-2.0811411819,1.2290590575
C,0.5049391798,-2.4538104055,0.0019479581
O,-0.6840469792,-3.0818632317,-2.0090214934
F,-0.2482728107,1.4495343648,-2.6946406387
F,0.4421883653,3.7232198612,-1.3745643639
F,0.7326070894,3.6892796416,1.3366248542
F,0.2757515167,1.4140717756,2.715676302
C,-0.8243771248,-3.1998935281,2.0816854603
H,-1.4124079102,-2.806923047,2.9092443948
H,-1.4413612225,-3.8770387307,1.4808707169
H,0.0075468724,-3.7953580915,2.4732532524
H,-1.1510839373,-0.7083673971,-2.3208981873
H,1.4029815962,-1.8274171805,-0.0436158448
H,0.7954893645,-3.5019026121,0.0072934253

01_b, Total Energy= -969.022080828 Hartree, NIMAG= 0
C,-0.3130993104,0.2452535536,-0.7482472076
C,-0.4060942491,0.2083906012,0.6522884727
C,-0.127257612,1.3739977228,1.3701652762
C,0.2114673906,2.5715578018,0.760707792
C,0.3043620682,2.6109774772,-0.6231030138
C,0.0557571006,1.4544716837,-1.3469367584
N,-0.7976576388,-0.9244109416,1.3820941102
N,-0.6413805271,-0.8260620791,-1.5863848362
C,-0.3349394946,-2.1870645563,-1.4267807775
C,-0.4484487098,-2.2407594636,1.1185891479
C,-0.1612436071,-2.7639176224,-0.0943179968
O,-0.2879310558,-2.881550215,-2.4281451161
F,0.1341939149,1.5080193321,-2.6915065649
F,0.6300603904,3.7458951196,-1.2485229443
F,0.4515806573,3.6643553713,1.4924170679
F,-0.2204993113,1.3342867941,2.7181205076
C,-0.4397486366,-3.101221436,2.3541477713
H,0.3091325855,-2.7465968849,3.0702285876

H,-1.4161791691,-3.0666966512,2.8493604305
H,-0.2221691078,-4.1382932608,2.1066225591
H,-0.6997940669,-0.5909844093,-2.5697087665
H,0.1059571339,-3.8103680276,-0.1364539191
H,-0.9569070008,-0.7247199116,2.3587814736

01_c, Total Energy= -968.991195094 Hartree, NIMAG= 0
C,-0.4265704092,0.2211349154,-0.6802236759
C,-0.2955088181,0.1725614387,0.7218158366
C,0.1360294907,1.3449294964,1.3616423505
C,0.4021290438,2.5213649558,0.6705088506
C,0.2417717615,2.557855367,-0.7061071534
C,-0.1610279842,1.4037900635,-1.3630645182
N,-0.6300118571,-0.8969847254,1.5567837734
N,-0.8630587484,-0.8964638478,-1.4313985928
C,-0.3070283041,-2.1437850179,-1.2321457782
C,-0.4611536418,-2.1354955246,1.2448628697
C,-0.0174794993,-2.6944705377,-0.0296749314
O,-0.1394479907,-2.7771431221,-2.4178370131
F,-0.3133211241,1.4342854761,-2.7066654789
F,0.4732511535,3.6832610759,-1.3901902868
F,0.7970146541,3.6184420613,1.3274981728
F,0.2886159345,1.3603168795,2.6899243052
C,-0.7728247335,-3.1518079695,2.3164914212
H,-1.0666613049,-2.646862677,3.2348220662
H,-1.5754489845,-3.8220903721,1.9923246585
H,0.1069464255,-3.7746479546,2.5126771857
H,-1.0511481466,-0.6976671115,-2.4052403025
H,0.4050403999,-3.6931662778,-0.0205265849
H,0.0843873341,-3.7029555774,-2.2660741873

01_d, Total Energy= -969.008049064 Hartree, NIMAG= 0
C,-0.2582656636,0.2052778435,-0.7158953433
C,-0.1943447597,0.1919620572,0.7063853871
C,0.0936591527,1.3988483282,1.3738777984
C,0.3357158914,2.5736746378,0.6913396817
C,0.2614429895,2.587834576,-0.7021212082
C,-0.0441936284,1.4264196331,-1.3828777381
N,-0.5509944133,-0.8846944405,1.5139403531
N,-0.6361422612,-0.8723326291,-1.5064486942
C,-0.3143314535,-2.0661490464,-1.1945405219
C,-0.283892956,-2.0970716688,1.2111917124
C,0.5330716109,-2.4605455348,-0.0215860267
O,-0.7776624806,-3.1012010797,-1.919718346
F,-0.1170103224,1.4721798366,-2.7202296482
F,0.4950322924,3.7253054961,-1.365464339

F,0.6403606754,3.6977529406,1.3496805029
F,0.1511563914,1.4170671466,2.7115238366
C,-0.8021915977,-3.214912713,2.0659059616
H,-1.3877574645,-2.8179956841,2.8933498909
H,-1.4233920818,-3.8932035332,1.4709247982
H,0.0297027886,-3.8099957349,2.4586572299
H,1.454032335,-1.8667118981,-0.0323195449
H,0.7810148887,-3.5198160358,-0.0577934277
H,-1.3460273066,-2.734319488,-2.614669301

01_e, Total Energy= -969.004546282 Hartree, NIMAG= 0
C,-0.2825738638,0.2051102916,-0.7723997426
C,-0.4533366085,0.190411577,0.6280430585
C,-0.1986818391,1.3473554343,1.3592007544
C,0.2122959309,2.5319289198,0.7630526724
C,0.3975136294,2.5557726777,-0.6089598265
C,0.1546969239,1.4047073667,-1.3514532568
N,-0.9103706346,-0.9428076818,1.3411921825
N,-0.5669528535,-0.835122335,-1.6545407617
C,-0.4550647799,-2.0762507216,-1.3564671807
C,-0.4478991967,-2.2376534244,1.1226540909
C,-0.1714319498,-2.7483198442,-0.0959133325
O,-0.6480858428,-2.9678328044,-2.3558562254
F,0.3373508539,1.4738081757,-2.6756094573
F,0.7979729126,3.6799895681,-1.2145351103
F,0.4266567396,3.6257605713,1.5026356995
F,-0.3766626335,1.3238692189,2.7019745174
C,-0.3391407572,-3.066166873,2.3712645102
H,0.4086141198,-2.644703497,3.0512734801
H,-1.2975941142,-3.0796111083,2.9016936361
H,-0.0638723967,-4.0937754022,2.1398711582
H,0.1465234271,-3.778711698,-0.1683170535
H,-1.0996451405,-0.7344446364,2.3109656057
H,-0.81155773,-2.4487514653,-3.1581868424

02_a, Total Energy= -1008.33819155 Hartree, NIMAG= 0
C,-0.2626495523,0.235267469,-0.725667463
C,-0.2910134594,0.222415119,0.6937287612
C,0.0332261367,1.405447227,1.3789172914
C,0.3840532337,2.5674769607,0.7159160523
C,0.4202714113,2.5763404079,-0.6748886092
C,0.113439413,1.421449647,-1.3733793419
N,-0.7008565473,-0.8593495379,1.467175221
N,-0.651742922,-0.8800653354,-1.5002363367
C,-0.2092468118,-2.1706620271,-1.2572271718
C,-0.3185589706,-2.0466363478,1.1772998156

C,0.6102813274,-2.3413079897,0.0153556245
O,-0.5212686108,-3.1038140247,-1.9703205818
F,0.2080148225,1.4567920243,-2.7150745459
F,0.7799111517,3.6856904878,-1.3277738918
F,0.6984977635,3.6731379793,1.3969208518
F,0.0129517484,1.4209501079,2.7162917148
C,-0.8352459418,-3.2076450442,1.9706683654
H,-1.5225269595,-2.8658269959,2.7427517751
H,-1.3453221185,-3.9174025888,1.3101257024
H,-0.0036533821,-3.7498122735,2.4336440939
C,-1.5603527869,-0.697968387,-2.6480327068
H,-2.1416545761,0.2096111777,-2.4976739497
H,-1.0125631543,-0.6296147668,-3.5884243685
H,-2.2241495061,-1.5598697608,-2.6912886754
H,1.4540513896,-1.6440374418,0.0238404576
H,0.9813334094,-3.3632103273,0.0361755986

02_b, Total Energy= -1008.33112530 Hartree, NIMAG= 0
C,-0.3509244931,0.2121411838,-0.7692716756
C,-0.555886564,0.1856635641,0.621325183
C,-0.2623446989,1.320644854,1.3786770251
C,0.2301383251,2.4833494689,0.8097304572
C,0.4828799573,2.5027885762,-0.5543955342
C,0.2191552415,1.3710539851,-1.315323617
N,-1.0275955511,-0.9594314443,1.2824011366
N,-0.7618680075,-0.8474260871,-1.6084116114
C,-0.3645723606,-2.1754996674,-1.4336168687
C,-0.4281893262,-2.2107281639,1.0954991565
C,0.0032541782,-2.669530343,-0.096358793
O,-0.4217659229,-2.9519513216,-2.3747417728
F,0.5115907938,1.4270995367,-2.6249499523
F,0.9907319788,3.5988296662,-1.1281484922
F,0.4867661508,3.5561036896,1.5638674018
F,-0.473900427,1.283202817,2.7129482672
C,-0.3231068981,-3.0276989114,2.3536815756
H,0.2994775048,-2.5223294527,3.0997199816
H,-1.3154070118,-3.1741258336,2.794874806
H,0.1017021756,-4.0087197259,2.1478371007
C,-1.4336761447,-0.535342332,-2.8812273843
H,-1.9703001112,0.4064415384,-2.7704975728
H,-0.733392584,-0.462145658,-3.7140757268
H,-2.1412858984,-1.3345911167,-3.0947722063
H,0.4650506074,-3.646748968,-0.127948534
H,-1.2872700479,-0.7746889392,2.2411950572

02_c, Total Energy= -1008.30022956 Hartree, NIMAG= 0
C,-0.3297740505,0.2344278902,-0.7309287411
C,-0.3618800313,0.2032011308,0.6789403473
C,0.0238922017,1.3654514285,1.3664853656
C,0.4280507721,2.516129507,0.7047282939
C,0.469692442,2.5315575191,-0.681888271
C,0.1123633579,1.3878264919,-1.3824107037
N,-0.7714995436,-0.8709226337,1.4679404762
N,-0.749594011,-0.8902263549,-1.4929365142
C,-0.1338205025,-2.0981788584,-1.2671562559
C,-0.4857030994,-2.0967734004,1.1806119579
C,0.1318911114,-2.6091504758,-0.0361111439
O,0.1305183958,-2.7623400837,-2.4216565006
F,0.1972495192,1.4196215917,-2.726814156
F,0.8598036199,3.6329773607,-1.3351360553
F,0.7790182598,3.6072535321,1.3948390663
F,0.0072433877,1.3897449882,2.7043634133
C,-0.8447064306,-3.1373161951,2.2141273463
H,-1.2727131452,-2.657594231,3.0926404367
H,-1.5570853039,-3.8614933946,1.8063043425
H,0.0492050405,-3.6975156258,2.5099489448
C,-1.648966344,-0.7255130784,-2.6435256254
H,-2.308263483,0.1184715615,-2.4407344618
H,-1.1151687158,-0.5464318456,-3.5780565683
H,-2.2587728587,-1.6237903482,-2.7465003133
H,0.6705258679,-3.5488179458,0.0259780357
H,0.3894109995,-3.6691902742,-2.2180232432

03_a, Total Energy= -571.984544062 Hartree, NIMAG= 0
C,-0.3196635463,0.2349820746,-0.6786252296
C,-0.2331221217,0.216374162,0.7340609317
C,0.1154457965,1.409494063,1.3931096271
C,0.3854541743,2.5756658609,0.6970288761
C,0.2784373076,2.5895104284,-0.6969810199
C,-0.0716821216,1.4301422983,-1.3709352826
N,-0.5829711517,-0.8715310627,1.5414480589
N,-0.7227888828,-0.8912235401,-1.4260764747
C,-0.3583684726,-2.2077695238,-1.2449172106
C,-0.3060205491,-2.0783295178,1.2242181769
C,0.4960043638,-2.4483538833,-0.0102956726
O,-0.7189257789,-3.0865960509,-2.0040430837
C,-0.7954466478,-3.2046078765,2.0876656367
H,-1.3811696799,-2.8177295403,2.9201442535
H,-1.4090492672,-3.894321581,1.4976705361
H,0.0493418106,-3.7857844156,2.4737970999
H,-0.1482194846,1.4309650194,-2.4534691015
H,0.4694931004,3.4990408389,-1.2542252958

H,0.6584676123,3.476536003,1.2337808009
H,0.1489633355,1.3848971242,2.4757703174
H,-1.2022494941,-0.7184241837,-2.3005352355
H,1.388853199,-1.8150788559,-0.0643459067
H,0.792412484,-3.4950413177,-0.006661741

03_b, Total Energy= -571.978955327 Hartree, NIMAG= 0
C,-0.3128381923,0.2572007452,-0.7424854492
C,-0.4187373718,0.2212003025,0.6567126624
C,-0.1409368213,1.3768691876,1.3932874727
C,0.2166908668,2.5685760588,0.7720773481
C,0.3291742684,2.6057144065,-0.6145529565
C,0.0779415283,1.4548736946,-1.3535834443
N,-0.8342835542,-0.932758182,1.3610092207
N,-0.658161964,-0.8325889555,-1.5674828136
C,-0.3181478012,-2.1804251761,-1.42435468
C,-0.4524259833,-2.2385923681,1.1119693798
C,-0.1281181916,-2.7578220148,-0.0949179877
O,-0.2589361839,-2.874542502,-2.4304842036
C,-0.4467935553,-3.1011786157,2.3474682692
H,0.2907041631,-2.7395300084,3.0722769012
H,-1.4288924518,-3.0788479912,2.8326072795
H,-0.2152037474,-4.1359600302,2.1020508627
H,0.1668984655,1.4775769242,-2.4347850824
H,0.6140619423,3.5196573284,-1.1217254955
H,0.412477485,3.4514063173,1.3684156305
H,-0.2205095348,1.3360728412,2.4754839106
H,-0.7514334061,-0.6036117871,-2.5488873689
H,0.1668565793,-3.7969847168,-0.133696874
H,-1.0404314836,-0.7446106247,2.3307815238

03_c, Total Energy= -571.947775116 Hartree, NIMAG= 0
C,-0.4202340646,0.2344082371,-0.6863824108
C,-0.3085231064,0.1886541975,0.7166421326
C,0.1078015378,1.356997014,1.37102365
C,0.406464004,2.5264658276,0.6792715769
C,0.2775437187,2.5575046908,-0.7060471894
C,-0.127861547,1.4079772515,-1.3805678166
N,-0.6347028159,-0.8955737375,1.5538454167
N,-0.8750277463,-0.8964470136,-1.4281281068
C,-0.3303931137,-2.1431202064,-1.2350122314
C,-0.4594716596,-2.1320867295,1.2428775355
C,-0.0296933566,-2.6956219853,-0.0355589184
O,-0.1748361771,-2.7864748139,-2.4231364704
C,-0.7431303479,-3.152054394,2.3220706767
H,-1.0342164629,-2.6482309893,3.2422324576

H,-1.5402938498,-3.8354206216,2.0112826503
H,0.1474392055,-3.7620321216,2.5116610294
H,-0.2248374145,1.4197028102,-2.4620831395
H,0.4874420762,3.4632855982,-1.2626825387
H,0.7191700046,3.4110359784,1.2218196686
H,0.1687406392,1.3188349448,2.4520821876
H,-1.0848276021,-0.706412526,-2.3983784648
H,0.3944493272,-3.6939019442,-0.03025549
H,0.0321062739,-3.714285242,-2.2615201282

03_d, Total Energy= -571.966019867 Hartree, NIMAG= 0
C,-0.2719310373,0.2191812405,-0.7179187494
C,-0.2223350875,0.2051729624,0.7033692912
C,0.0701752633,1.4061852284,1.3796486899
C,0.3592074763,2.5730735592,0.6958195017
C,0.3026485657,2.5870729438,-0.7038895263
C,-0.0332792465,1.4334379508,-1.3895871739
N,-0.5712448895,-0.884120405,1.5130082476
N,-0.6431077472,-0.8697668828,-1.514832054
C,-0.3207891993,-2.0603798107,-1.2015076398
C,-0.2943719384,-2.0936448822,1.2116269227
C,0.5199609624,-2.453786805,-0.0238231097
O,-0.7687954976,-3.1024980583,-1.9378793691
C,-0.7874161493,-3.2180562606,2.07611627
H,-1.3721182089,-2.8263473192,2.9070781286
H,-1.4049423413,-3.9081675081,1.4907795325
H,0.0554260743,-3.8002726404,2.4655958529
H,-0.1146266588,1.4293860368,-2.4702851687
H,0.5026715008,3.5017710352,-1.2502284278
H,0.6033905622,3.4762607905,1.2430709237
H,0.0612520796,1.3802431897,2.4630741048
H,1.4396831101,-1.8573758904,-0.0323996499
H,0.7713617322,-3.5125871939,-0.0603016349
H,-1.3372363027,-2.7325112433,-2.6302871582

03_e, Total Energy= -571.960184604 Hartree, NIMAG= 0
C,-0.2752118283,0.2202646339,-0.7649938655
C,-0.4469270845,0.2021916658,0.6344412764
C,-0.1884732341,1.352512366,1.3807896622
C,0.2259777977,2.5359335734,0.7725573823
C,0.404140005,2.565038205,-0.6058261505
C,0.1520125635,1.4178170857,-1.3531171043
N,-0.9207048353,-0.9471823888,1.3332565246
N,-0.5418043967,-0.8367809571,-1.6513873074
C,-0.4245298411,-2.0747997267,-1.3530807156
C,-0.4622059094,-2.2388844491,1.1234031326

C,-0.1620162541,-2.7505206217,-0.0904363298
O,-0.5868119919,-2.9720739339,-2.3626353454
C,-0.3765058232,-3.0745474075,2.3709177283
H,0.365989228,-2.6623243401,3.0627784844
H,-1.3420851105,-3.0840252619,2.8887981086
H,-0.1054450468,-4.1034603495,2.1395587392
H,0.2630470251,1.4228205896,-2.4308099167
H,0.7245998057,3.4739520959,-1.1014084826
H,0.4051973397,3.4182247626,1.3755974274
H,-0.3248633723,1.3209140979,2.4580727709
H,0.1550457242,-3.781587958,-0.1579544718
H,-1.1354590792,-0.7455221266,2.2987828984
H,-0.743623588,-2.4468112071,-3.1614555976

04_a, Total Energy= -611.298907752 Hartree, NIMAG= 0
C,-0.285033527,0.2369460845,-0.7094232047
C,-0.2800386009,0.2255160664,0.7084312707
C,0.0528294859,1.4102915366,1.388880094
C,0.4047707423,2.566004709,0.7124809586
C,0.4016281216,2.5728459133,-0.6843639985
C,0.0542140769,1.423996061,-1.3793496644
N,-0.6674674213,-0.8610677312,1.5004277188
N,-0.6963383714,-0.8854057605,-1.4795843102
C,-0.2677922427,-2.1793313721,-1.2504653247
C,-0.316624259,-2.0536340953,1.2011706747
C,0.5652894742,-2.3662518158,0.0089677504
O,-0.5723934209,-3.1083739325,-1.9783382774
C,-0.8080215479,-3.2073614942,2.0248944369
H,-1.460495374,-2.8539819579,2.822035451
H,-1.3523038884,-3.9182428042,1.3934555611
H,0.0365054556,-3.7540372646,2.4589270227
C,-1.4945834393,-0.6610925056,-2.6916966639
H,-2.1510625089,0.1930403367,-2.532280814
H,-0.8647343221,-0.4795323122,-3.568058405
H,-2.0838106743,-1.5559375178,-2.8809271636
H,0.0539169296,1.4358501624,-2.4620940232
H,0.6675435896,3.4703318008,-1.2305863759
H,0.6662549547,3.4606653196,1.265352659
H,0.0147619235,1.385460603,2.4713993343
H,1.4213056198,-1.6831229262,-0.0050436618
H,0.9195062661,-3.3946269948,0.0193927381

04_b, Total Energy= -611.290329196 Hartree, NIMAG= 0
C,-0.3834816186,0.2263962225,-0.7447756947
C,-0.5196522379,0.1959504343,0.6533503703
C,-0.1990495726,1.3288742577,1.4071492246

C,0.2536794397,2.4960710926,0.8028628695
C,0.4233333083,2.5235264843,-0.577734598
C,0.1155783385,1.396729144,-1.3337402019
N,-0.9848949839,-0.9561540188,1.3304115926
N,-0.8093097408,-0.8510477598,-1.5715406119
C,-0.3805190526,-2.1724726117,-1.4097495344
C,-0.4468236986,-2.2205152351,1.1238887784
C,-0.0498541524,-2.6921465512,-0.0769611108
O,-0.3677879664,-2.9285094425,-2.3739117659
C,-0.353192217,-3.0581916156,2.3711624453
H,0.3177554323,-2.5968525583,3.1042446682
H,-1.3394750535,-3.1548851315,2.8389189342
H,0.0115000165,-4.0588252975,2.1450491877
C,-1.3283628919,-0.5243975863,-2.9037210763
H,-1.9260422168,0.3843491683,-2.8332470527
H,-0.5338743591,-0.3852692289,-3.6445844585
H,-1.9506524213,-1.3496011848,-3.242768628
H,0.2471333272,1.4285550791,-2.407118742
H,0.79215797,3.4154909321,-1.0701513448
H,0.4829109983,3.3653125481,1.4076327776
H,-0.3149904842,1.2874660816,2.4859904607
H,0.3754179355,-3.6854043031,-0.1175301579
H,-1.220450476,-0.7701694547,2.2946233526

04_c, Total Energy= -611.258661817 Hartree, NIMAG= 0
C,-0.3735805546,0.2361930266,-0.7106826469
C,-0.3437705437,0.1997979236,0.6976437679
C,0.0651454793,1.3577975174,1.3768046763
C,0.4509384152,2.5088388801,0.700549495
C,0.4214104483,2.5307144037,-0.6911031371
C,0.0151154682,1.3948691365,-1.3876160026
N,-0.7274280802,-0.8791685243,1.5126150311
N,-0.8340891662,-0.8987334044,-1.4606862056
C,-0.2166116908,-2.1084015431,-1.2467332471
C,-0.4789795005,-2.1081201505,1.2170033426
C,0.0723929745,-2.6315094385,-0.0270040861
O,0.0419527849,-2.7724952571,-2.4083511858
C,-0.7989535506,-3.1448964686,2.2694392517
H,-1.1747565521,-2.6582688839,3.1681732161
H,-1.5434495785,-3.8582008588,1.9010437214
H,0.0994973394,-3.71971265,2.521223966
C,-1.5507228256,-0.6761412365,-2.7171209568
H,-2.2388025836,0.1569154569,-2.5724225565
H,-0.8891160023,-0.4527142834,-3.5610834759
H,-2.1260429246,-1.5674886265,-2.9651730342
H,-0.0067501976,1.4134280546,-2.4702176781
H,0.705422559,3.4231287528,-1.2367114498

H,0.7568079154,3.3870882407,1.2574872425
H,0.0536543731,1.3238191381,2.4596934763
H,0.5991281231,-3.5795024751,0.0103272184
H,0.3126786315,-3.6744956578,-2.2007251579

05_a, Total Energy= -763.767296058 Hartree, NIMAG= 0
C,-0.134183485,0.3693052351,-0.4356126979
C,0.0342411033,0.2390396108,0.966278475
C,0.5229716415,1.3517829034,1.679002863
C,0.8516992574,2.538239649,1.0468648291
C,0.6635369404,2.6623259714,-0.3334001072
C,0.1739430589,1.5879717041,-1.0590177289
N,-0.3626250026,-0.8646547456,1.7169311398
N,-0.6788750968,-0.6595284104,-1.2313282387
C,-0.4146790815,-2.0103239124,-1.1653980994
C,-0.2370523808,-2.074507835,1.3046278449
C,0.4725231964,-2.4021259957,0.0033892737
O,-0.8778468716,-2.799778639,-1.9660143407
H,1.2339158963,3.3719436676,1.6239939404
H,0.620011366,1.2469547473,2.7529617432
H,0.8993948552,3.591052981,-0.8393891399
H,0.0323528657,1.6743796703,-2.1315037788
C,-0.794033068,-3.1731004002,2.1330257826
C,-1.0648262871,-4.438211849,1.5909649995
C,-1.085038434,-2.9436575611,3.4881739481
C,-1.6139113138,-5.4450089593,2.3826510103
H,-0.8816344843,-4.6380150518,0.5423316767
C,-1.6173602827,-3.9540762129,4.2777262994
H,-0.8842775127,-1.9640643284,3.9021317383
C,-1.8851882276,-5.2096506691,3.7278021147
H,-1.8304425949,-6.4124935108,1.9443092819
H,-1.8262641749,-3.7649772547,5.3247667018
H,-2.3039527751,-5.996636021,4.3449400919
H,-1.1835864806,-0.3822383394,-2.0637600396
H,1.3937874868,-1.8105645131,-0.0376254493
H,0.7262275371,-3.4529602337,-0.09193956

05_b, Total Energy= -763.760975552 Hartree, NIMAG= 0
C,-0.0008333123,0.4157699804,-0.5302505153
C,-0.338764048,0.2448547341,0.8210835909
C,-0.0791196819,1.2772267518,1.7278561082
C,0.4920572185,2.4775096499,1.3187251811
C,0.8402077168,2.6449815508,-0.0184969174
C,0.6044964105,1.6155369543,-0.923314745
N,-0.9744662138,-0.9220118747,1.3023478435
N,-0.3192918305,-0.5321752223,-1.5245216626

C,-0.139060496,-1.9175510755,-1.4994568892
C,-0.688421144,-2.2293758429,0.961750983
C,-0.2075836243,-2.6488553113,-0.2365804077
O,0.0076007632,-2.5046662686,-2.563533609
H,0.6691261137,3.2655656062,2.0405630629
H,-0.3443723669,1.1334189381,2.7707369924
H,1.2946730102,3.5675061153,-0.359325331
H,0.8735566168,1.7413210524,-1.9669401882
C,-0.9600997509,-3.2184659875,2.0430919309
C,-1.6140509636,-4.4237256612,1.7547227476
C,-0.5629685523,-2.9659699102,3.3643939972
C,-1.8592449678,-5.3537887094,2.7608839015
H,-1.9435920019,-4.6185047193,0.7412189579
C,-0.8043498671,-3.8999013974,4.3685751667
H,-0.0272046944,-2.0533465491,3.6023578675
C,-1.4554516024,-5.0956124119,4.0700417204
H,-2.3736179979,-6.2779909632,2.5231987329
H,-0.4752842761,-3.6975541607,5.3815351921
H,-1.6467068353,-5.8212783099,4.8522940948
H,-0.237487562,-0.1904187819,-2.4736363962
H,0.0396388249,-3.6949791785,-0.3442354606
H,-1.4154328161,-0.809986683,2.2023843788

05_c, Total Energy= -763.730493019 Hartree, NIMAG= 0
C,-0.214873451,0.393159881,-0.4899750914
C,-0.1586898612,0.1938736753,0.9036880037
C,0.3224256141,1.2523381891,1.690076124
C,0.7413590545,2.4556171862,1.1327021262
C,0.6681595552,2.6381316744,-0.2453039817
C,0.1968961774,1.6018499259,-1.0486892959
N,-0.6088674502,-0.9348858605,1.6021443518
N,-0.7326812609,-0.6198290875,-1.3523799
C,-0.2497391448,-1.906192481,-1.2758369495
C,-0.5256192792,-2.1509459467,1.1771064465
C,-0.0584885458,-2.6054687971,-0.1325697803
O,-0.0246199923,-2.4126436456,-2.5175851113
H,1.1036766759,3.2511275689,1.7733602702
H,0.3388013061,1.1022901751,2.7629047273
H,0.9708541957,3.5754258654,-0.6971343893
H,0.1412163621,1.7325333852,-2.1251759539
C,-0.9481265584,-3.213915055,2.1428242909
C,-1.3987500342,-4.4684755082,1.7098500953
C,-0.9088588398,-2.9574701011,3.521468188
C,-1.7994775512,-5.4387055248,2.6267217114
H,-1.4656159424,-4.6806776439,0.6495509276
C,-1.3001120423,-3.9277686386,4.4363295943
H,-0.5674725158,-1.9858105722,3.853785943

C,-1.7474002962,-5.1736180115,3.9929959858
H,-2.1570981847,-6.3990478297,2.2720281802
H,-1.2555073152,-3.7146265537,5.498717507
H,-2.0536402685,-5.9297575759,4.7073430668
H,-0.8877791897,-0.3179198571,-2.3046684816
H,0.3618992507,-3.6005332602,-0.2099278696
H,0.143900431,-3.3601771631,-2.4542035742

05_d, Total Energy= -763.748846940 Hartree, NIMAG= 0
C,-0.0932092208,0.3615389725,-0.4766049973
C,0.0517941987,0.2252139701,0.9342365919
C,0.5042288527,1.3389298375,1.6735841473
C,0.8581751061,2.5250245767,1.0587801533
C,0.7073117661,2.6585373088,-0.3282113237
C,0.2145031337,1.6005652008,-1.0704013109
N,-0.3461406767,-0.8823540407,1.6813697471
N,-0.6223753809,-0.6175569919,-1.32339512
C,-0.3987619104,-1.8540219576,-1.1212237424
C,-0.2334279958,-2.0951639637,1.276987908
C,0.4722094052,-2.4090109065,-0.0351452466
O,-0.9873658314,-2.7896026209,-1.8983646416
H,1.2261588669,3.354438337,1.6516698365
H,0.5683552918,1.2267159306,2.7495963304
H,0.9572440816,3.5927188771,-0.8180174168
H,0.0582215191,1.6924377277,-2.1388730541
C,-0.7925435247,-3.188631284,2.1115960604
C,-0.9987322602,-4.4812371137,1.6076434942
C,-1.1443410162,-2.9265133605,3.4470536629
C,-1.5395290801,-5.481419056,2.413769695
H,-0.7664668627,-4.714602784,0.5762219349
C,-1.6711879126,-3.9279003639,4.2511640432
H,-0.9933464118,-1.926828254,3.8329274393
C,-1.8722681551,-5.2113036115,3.7380911697
H,-1.7011627449,-6.4713731591,2.002509162
H,-1.9279717844,-3.7096862844,5.2817986009
H,-2.2864483911,-5.9916600327,4.3665612488
H,1.4239617824,-1.8665061291,-0.0412812895
H,0.6678629825,-3.466694874,-0.1843894588
H,-1.5682500381,-2.319639366,-2.515775959

05_e, Total Energy= -763.742077162 Hartree, NIMAG= 0
C,0.0407679971,0.3715438245,-0.5482422387
C,-0.3745403284,0.2206555211,0.790418056
C,-0.1166443626,1.2316964134,1.7168338323
C,0.5415212146,2.4034974855,1.3473089079
C,0.9662868622,2.5590238221,0.0327164967

C,0.7117063431,1.5514932866,-0.8939446527
N,-1.0997878688,-0.9213292374,1.2368207306
N,-0.2018987383,-0.5292528317,-1.5993262173
C,-0.2898326671,-1.797141401,-1.4560276095
C,-0.7721887744,-2.2340729532,0.9404013005
C,-0.3203346419,-2.6408084229,-0.2705572278
O,-0.3872273337,-2.5428152182,-2.5901356513
H,0.7148344859,3.1797819711,2.0832556435
H,-0.4487594702,1.1014239636,2.7427481014
H,1.4794602245,3.4617647343,-0.2774262526
H,1.0106763602,1.6623461708,-1.9293658761
C,-0.9865214757,-3.2115212423,2.0413434264
C,-1.5806943789,-4.4551873476,1.786721795
C,-0.5970610205,-2.9064204354,3.3540036332
C,-1.7709556848,-5.3724173283,2.8161581738
H,-1.9113723577,-4.6893433269,0.7819004261
C,-0.7830396922,-3.8275169898,4.3812547128
H,-0.1117804453,-1.959912264,3.5658127417
C,-1.3723279816,-5.0625741509,4.1157265879
H,-2.2404252682,-6.3265025752,2.6049073884
H,-0.4605635917,-3.5835598193,5.387142629
H,-1.5220240279,-5.7776926396,4.9165472078
H,-0.064485724,-3.68068297,-0.4130548796
H,-1.5404006343,-0.7950106299,2.135254236
H,-0.3407648124,-1.9139402071,-3.3257320019

06_a, Total Energy= -803.081735507 Hartree, NIMAG= 0
C,-0.1084098506,0.3672377183,-0.4584868833
C,0.0073224907,0.2585007471,0.9529359582
C,0.5045798782,1.3631877663,1.670860303
C,0.9130072108,2.523516087,1.0369567259
C,0.8015944159,2.6238951565,-0.3521058175
C,0.29073186,1.5609320663,-1.0817006336
N,-0.4221442948,-0.8305984311,1.7074042186
N,-0.688054675,-0.6550944051,-1.25839997
C,-0.3704996693,-1.9953375536,-1.1453481635
C,-0.2434614767,-2.039609335,1.3120165102
C,0.5189778347,-2.3411300374,0.0368597929
O,-0.8068299035,-2.8396846486,-1.9087245082
H,0.5505969679,1.2704732042,2.749338986
H,1.302545243,3.3513630076,1.6178084805
H,1.1095650867,3.5276353693,-0.8648445174
H,0.2058558781,1.6458070201,-2.1577784473
C,-0.8001503245,-3.1510488603,2.1219690461
C,-1.008133345,-4.425662356,1.5746371866
C,-1.1527651744,-2.9261347793,3.4630635948
C,-1.5552183469,-5.4472631289,2.3486447108

H,-0.7775425153,-4.6202929317,0.5344587742
C,-1.6838580461,-3.9506179531,4.2352426898
H,-0.9993842222,-1.9392342557,3.8801343806
C,-1.8881130698,-5.2161871296,3.6807105651
H,-1.7223849685,-6.4228905242,1.9066655243
H,-1.9406785706,-3.7651259875,5.2722458522
H,-2.3056602027,-6.0144082002,4.2841333413
C,-1.5479274559,-0.2748321136,-2.3866117678
H,-2.1060688841,0.6220473026,-2.1220218183
H,-0.9704553455,-0.0908241106,-3.2978238751
H,-2.232877973,-1.0970584311,-2.5830809831
H,1.4193658876,-1.7183773485,0.0175359705
H,0.8102040871,-3.3830550956,-0.0510137089

06_b, Total Energy= -803.072449785 Hartree, NIMAG= 0
C,-0.1914102252,0.379409864,-0.5213040848
C,-0.4931780981,0.2086523126,0.8399028401
C,-0.1075701874,1.1832434832,1.765083778
C,0.578077523,2.3253729041,1.3673089553
C,0.9159015578,2.4824529199,0.0266115136
C,0.5401373875,1.5140611769,-0.8990790172
N,-1.1931665374,-0.928683103,1.3035066996
N,-0.6786753338,-0.5097244835,-1.5209234971
C,-0.4767625335,-1.8921238774,-1.499055354
C,-0.8269334759,-2.2294806917,0.9887865323
C,-0.3566594705,-2.6082340427,-0.223568973
O,-0.48367499,-2.5250707856,-2.5486996292
H,-0.358403249,1.0382305993,2.811354381
H,0.8552426155,3.0735556532,2.1003288079
H,1.4671255257,3.3548412646,-0.3039475841
H,0.7997052334,1.6489494246,-1.9407704745
C,-0.986371352,-3.2193077312,2.0888771409
C,-1.5239525037,-4.4874519409,1.8289799283
C,-0.6007992081,-2.9083051396,3.4015175605
C,-1.6638606668,-5.4208165363,2.8518725461
H,-1.8483401729,-4.7286911281,0.8238760289
C,-0.7381685861,-3.8444062486,4.4230396389
H,-0.1540429544,-1.9443440259,3.6200519775
C,-1.2717262675,-5.1031385062,4.1515443339
H,-2.08854224,-6.3944790872,2.635271332
H,-0.4196347957,-3.5938576137,5.4286011154
H,-1.3821980775,-5.8308133358,4.9474344842
C,-1.0019630109,0.0535846807,-2.8357756885
H,-1.4608692414,1.0319871939,-2.6941266759
H,-0.122575719,0.1503183528,-3.4814152709
H,-1.7018889745,-0.6135109161,-3.3340473686
H,-0.0300440705,-3.6307517262,-0.348294262

H,-1.5909716981,-0.8193558268,2.2242788554

06_c, Total Energy= -803.041705448 Hartree, NIMAG= 0
C,-0.2148794501,0.3857131758,-0.4838195202
C,-0.1643777529,0.2011250396,0.9134276436
C,0.354507444,1.2453137108,1.6963356599
C,0.8310766396,2.4207296103,1.1291990488
C,0.7805659645,2.5877505438,-0.2521548073
C,0.2633354924,1.5692536851,-1.0498205771
N,-0.6389992411,-0.9129824915,1.6146370153
N,-0.7885709717,-0.6259367701,-1.3256075659
C,-0.2441620302,-1.8881812522,-1.2517360085
C,-0.5228108382,-2.1303260506,1.1979952304
C,-0.0118352899,-2.565097136,-0.0986445211
O,0.0100415911,-2.4211308913,-2.4799015883
H,0.3568702642,1.104838263,2.7706387149
H,1.2228933209,3.2075081038,1.7635723281
H,1.1329427899,3.5039896704,-0.7115490456
H,0.2245986633,1.7014259138,-2.1239423339
C,-0.9470943552,-3.2014043407,2.1530863187
C,-1.3345716454,-4.4726539737,1.7080928044
C,-0.9746180444,-2.9383981909,3.5308644637
C,-1.7379860397,-5.4536498241,2.6121359999
H,-1.3478935075,-4.6896486589,0.6467451174
C,-1.371093423,-3.9182698238,4.4332544403
H,-0.6815025736,-1.9539889409,3.871773472
C,-1.7541336593,-5.1812024434,3.9779417523
H,-2.0449305046,-6.4279283273,2.248017288
H,-1.3800424506,-3.699656081,5.4954598387
H,-2.0634660401,-5.944962829,4.6828244433
C,-1.4888506658,-0.2238536672,-2.5458244762
H,-2.1139961051,0.6377405981,-2.3104125797
H,-0.8150171056,0.0352014993,-3.3696641036
H,-2.1292756857,-1.041296678,-2.8760129009
H,0.4884044753,-3.5245276147,-0.1586232614
H,0.2394801866,-3.3525071823,-2.3788347072

****
01new_3H_4Fs_2oxo_4me_giao
****

Atom	Abs.	Rel.
1C	61.84	116.15
2C	51.06	126.53
3C	31.62	145.25
4C	37.25	139.83
5C	36.35	140.70

6C	38.05	139.06
7N	-73.99	-82.01
8N	110.71	-256.73
9C	14.90	161.35
10C	10.26	165.82
11C	136.22	44.52
12H	28.63	3.23
13H	29.40	2.49
14O	-89.28	330.18
15H	25.05	6.71
16F	334.86	-159.03
17F	335.82	-159.95
18F	338.95	-162.95
19F	317.94	-142.81
20C	153.48	27.90
21H	29.57	2.32
22H	29.64	2.25
23H	29.52	2.36

****
02new_3H_4Fs_1me_2oxo_4me_giao
****

Atom	Abs.	Rel.
1C	55.58	122.17
2C	47.68	129.79
3C	33.08	143.85
4C	36.34	140.70
5C	36.62	140.43
6C	34.20	142.77
7N	-70.85	-84.97
8N	110.17	-256.22
9C	13.90	162.32
10C	7.09	168.87
11C	136.62	44.13
12H	28.66	3.20
13H	29.41	2.47
14O	-88.49	329.46
15F	321.38	-146.10
16F	336.63	-160.72
17F	336.32	-160.43
18F	319.52	-144.32
19C	154.04	27.36
20H	29.64	2.25
21H	29.65	2.24
22H	29.57	2.32
23C	144.14	36.89
24H	29.55	2.34

25H	28.44	3.41
26H	28.20	3.65

****
03new_3H_2oxo_4me_giao
****

Atom	Abs.	Rel.
1C	47.34	130.11
2C	35.81	141.21
3C	48.21	129.28
4C	54.38	123.33
5C	52.62	125.02
6C	58.27	119.59
7N	-92.55	-64.45
8N	98.34	-245.03
9C	13.37	162.83
10C	13.83	162.38
11C	137.35	43.43
12H	28.74	3.12
13H	29.38	2.50
14O	-75.62	317.90
15H	25.14	6.62
16C	153.24	28.13
17H	29.65	2.24
18H	29.62	2.26
19H	29.55	2.34
20H	24.97	6.78
21H	24.69	7.05
22H	24.64	7.10
23H	24.33	7.40

****
04new_3H_1me_2oxo_4me_giao
****

Atom	Abs.	Rel.
1C	40.23	136.96
2C	33.89	143.06
3C	49.45	128.08
4C	54.31	123.40
5C	53.29	124.38
6C	57.85	119.99
7N	-90.08	-66.79
8N	102.37	-248.85
9C	12.52	163.65
10C	12.15	164.00
11C	137.07	43.71

12H	28.72	3.14
13H	29.39	2.49
14O	-81.97	323.60
15C	153.92	27.48
16H	29.73	2.16
17H	29.64	2.25
18H	29.59	2.29
19C	146.96	34.18
20H	29.41	2.48
21H	28.69	3.17
22H	27.72	4.11
23H	24.60	7.14
24H	24.61	7.13
25H	24.66	7.08
26H	24.39	7.34

****
05new_3H_2oxo_4ph_giao
****

Atom	Abs.	Rel.
1C	46.84	130.59
2C	35.68	141.34
3C	48.15	129.33
4C	54.16	123.55
5C	52.50	125.14
6C	58.16	119.69
7H	24.25	7.47
8H	24.60	7.14
9H	24.62	7.12
10H	24.89	6.85
11N	-89.09	-67.72
12N	96.75	-243.53
13C	12.31	163.84
14C	18.12	158.25
15C	140.73	40.18
16H	28.02	3.82
17H	29.30	2.58
18O	-73.54	316.04
19H	25.02	6.73
20C	38.06	139.05
21C	49.65	127.88
22C	48.71	128.80
23C	49.73	127.81
24H	23.75	7.96
25C	49.51	128.02
26H	23.06	8.64
27C	47.08	130.36

28H	24.29	7.44
29H	24.29	7.44
30H	24.33	7.40

****
06new_3H_1me_2oxo_4ph_giao
****

Atom	Abs.	Rel.
1C	39.49	137.67
2C	33.93	143.03
3C	49.45	128.08
4C	54.13	123.58
5C	53.28	124.39
6C	57.64	120.19
7H	24.29	7.43
8H	24.55	7.18
9H	24.55	7.19
10H	24.61	7.13
11N	-86.68	-70.01
12N	101.01	-247.55
13C	11.54	164.59
14C	16.47	159.84
15C	140.51	40.39
16H	27.98	3.86
17H	29.25	2.63
18O	-81.97	323.61
19C	38.28	138.84
20C	49.62	127.91
21C	49.24	128.28
22C	49.63	127.90
23H	23.70	8.01
24C	49.68	127.85
25H	23.04	8.65
26C	47.18	130.27
27H	24.28	7.45
28H	24.34	7.39
29H	24.33	7.40
30C	146.87	34.27
31H	29.41	2.47
32H	28.64	3.22
33H	27.72	4.12
